# Longer-term efficiency and safety of increasing the frequency of whole blood donation (INTERVAL): extension study of a randomised trial of 20 757 blood donors

**DOI:** 10.1016/S2352-3026(19)30106-1

**Published:** 2019-08-02

**Authors:** Stephen Kaptoge, Emanuele Di Angelantonio, Carmel Moore, Matthew Walker, Jane Armitage, Willem H Ouwehand, David J Roberts, John Danesh, Simon G Thompson, INTERVAL Trial Group, Stephen Kaptoge, Emanuele Di Angelantonio, Carmel Moore, Matthew Walker, Jane Armitage, Willem H Ouwehand, David J Roberts, John Danesh, Simon G Thompson, Jenny Donovan, Ian Ford, Rachel Henry, Beverley J Hunt, Bridget le Huray, Susan Mehenny, Gail Miflin, Jane Green, Mike Stredder, Nicholas A Watkins, Alan McDermott, Clive Ronaldson, Claire Thomson, Zoe Tolkien, Lorna Williamson, David Allan, Jennifer Sambrook, Tracey Hammerton, David Bruce, Fizzah Choudry, Cedric Ghvaert, Kirstie Jonston, Anne Kelly, Andrew King, Alfred Mo, Lizanne Page, Penny Richardson, Peter Senior, Yagnesh Umrania, Henna Wong, Brendan Burchell, John Gallacher, Gavin Murphy, Adrian C Newland, Keith Wheatley, Michael Greaves, Marc Turner, Tahir Aziz, Richard Brain, Christine Davies, Ruth Turner, Paula Wakeman, Alison Dent, Alan Wakeman, Ben Anthony, Desmond Bland, Willem H Parrondo, Helen Vincent, Candy Weatherill, Andrea Forsyth, Carol Butterfield, Tracey Wright, Karen Ellis, Kristie Johnston, Pat Poynton, Carolyn Brooks, Emma Martin, Lara Littler, Lindsay Williamson, Donna Blair, Karen Ackerley, Lynn Woods, Sophie Stanley, Gemma Walsh, Gayle Franklin, Cheryl Howath, Sarah Sharpe, Deborah Smith, Lauren Botham, Caroline Williams, Claire Alexander, Gareth Sowerbutts, Diane Furnival, Michael Thake, Shilpa Patel, Carolyn Roost, Sandra Sowerby, Mary Joy Appleton, Eileen Bays, Geoff Bowyer, Steven Clarkson, Stuart Halson, Kate Holmes, Gareth Humphreys, Lee Parvin-Cooper, Jason Towler, Joanne Addy, Patrica Barrass, Louise Stennett, Susan Burton, Hannah Dingwell, Victoria Clarke, Maria Potton, Thomas Bolton, Michael Daynes, Stuart Halson, Sarah Spackman, Michael Walker, Abudu Momodu, James Fenton, Adam King, Omer Muhammad, Nicholas Oates, Tim Peakman, Christine Ryan, Kristian Spreckley, Craig Stubbins, Joanna Williams, James Brannan, Cedric Mochon, Samantha Taylor, Kimberly Warren, Stephen Kaptoge, Emanuele Di Angelantonio, Jonathan Mant, Willem H Ouwehand, Simon G Thompson, John Danesh, David J Roberts

**Affiliations:** a Department of Public Health and Primary Care, Strangeways Research Laboratory, Cambridge, UK; b NIHR Blood and Transplant Research Unit in Donor Health and Genomics, Strangeways Research Laboratory, Cambridge, UK; c Clinical Trial Service Unit and Epidemiological Studies Unit, Nuffield Department of Population Health, and MRC Population Health Research Unit, University of Oxford, Oxford, UK; d Department of Haematology, University of Cambridge, Cambridge Biomedical Campus, Cambridge, UK; e NIHR Oxford Biomedical Research Centre—Haematology Theme and Radcliffe Department of Medicine, University of Oxford, John Radcliffe Hospital, Oxford, UK; f NHS Blood and Transplant, Cambridge, UK; g Oxford, UK; h NIHR Cambridge Biomedical Research Centre, Addenbrooke's Hospital, Cambridge, UK; i British Heart Foundation Cambridge Centre for Research Excellence, Addenbrooke's Hospital, Cambridge, UK

## Abstract

**Background:**

The INTERVAL trial showed that, over a 2-year period, inter-donation intervals for whole blood donation can be safely reduced to meet blood shortages. We extended the INTERVAL trial for a further 2 years to evaluate the longer-term risks and benefits of varying inter-donation intervals, and to compare routine versus more intensive reminders to help donors keep appointments.

**Methods:**

The INTERVAL trial was a parallel group, pragmatic, randomised trial that recruited blood donors aged 18 years or older from 25 static donor centres of NHS Blood and Transplant across England, UK. Here we report on the prespecified analyses after 4 years of follow-up. Participants were whole blood donors who agreed to continue trial participation on their originally allocated inter-donation intervals (men: 12, 10, and 8 weeks; women: 16, 14, and 12 weeks). They were further block-randomised (1:1) to routine versus more intensive reminders using computer-generated random sequences. The prespecified primary outcome was units of blood collected per year analysed in the intention-to-treat population. Secondary outcomes related to safety were quality of life, self-reported symptoms potentially related to donation, haemoglobin and ferritin concentrations, and deferrals because of low haemoglobin and other factors. This trial is registered with ISRCTN, number ISRCTN24760606, and has completed.

**Findings:**

Between Oct 19, 2014, and May 3, 2016, 20 757 of the 38 035 invited blood donors (10 843 [58%] men, 9914 [51%] women) participated in the extension study. 10 378 (50%) were randomly assigned to routine reminders and 10 379 (50%) were randomly assigned to more intensive reminders. Median follow-up was 1·1 years (IQR 0·7–1·3). Compared with routine reminders, more intensive reminders increased blood collection by a mean of 0·11 units per year (95% CI 0·04–0·17; p=0·0003) in men and 0·06 units per year (0·01–0·11; p=0·0094) in women. During the extension study, each week shorter inter-donation interval increased blood collection by a mean of 0·23 units per year (0·21–0·25) in men and 0·14 units per year (0·12–0·15) in women (both p<0·0001). More frequent donation resulted in more deferrals for low haemoglobin (odds ratio per week shorter inter-donation interval 1·19 [95% CI 1·15–1·22] in men and 1·10 [1·06–1·14] in women), and lower mean haemoglobin (difference per week shorter inter-donation interval −0·84 g/L [95% CI −0·99 to −0·70] in men and −0·45 g/L [–0·59 to −0·31] in women) and ferritin concentrations (percentage difference per week shorter inter-donation interval −6·5% [95% CI −7·6 to −5·5] in men and −5·3% [–6·5 to −4·2] in women; all p<0·0001). No differences were observed in quality of life, serious adverse events, or self-reported symptoms (p>0.0001 for tests of linear trend by inter-donation intervals) other than a higher reported frequency of doctor-diagnosed low iron concentrations and prescription of iron supplements in men (p<0·0001).

**Interpretation:**

During a period of up to 4 years, shorter inter-donation intervals and more intensive reminders resulted in more blood being collected without a detectable effect on donors' mental and physical wellbeing. However, donors had decreased haemoglobin concentrations and more self-reported symptoms compared with the initial 2 years of the trial. Our findings suggest that blood collection services could safely use shorter donation intervals and more intensive reminders to meet shortages, for donors who maintain adequate haemoglobin concentrations and iron stores.

**Funding:**

NHS Blood and Transplant, UK National Institute for Health Research, UK Medical Research Council, and British Heart Foundation.

## Introduction

INTERVAL was the first randomised trial, to the best of our knowledge, to evaluate the efficiency and safety of varying the frequency of whole blood donation.^1^, ^2^, ^3^ We randomly assigned over 45 000 blood donors recruited across England, UK, to different inter-donation intervals (8, 10, and 12 weeks for men; and 12, 14, and 16 weeks for women) over a period of 2 years with more intensive reminders than standard for NHS Blood and Transplant (NHSBT). During that time, there was a substantial increase in the amount of blood collected by reducing the inter-donation intervals combined with intensive reminders to follow up missed appointments without detectable effects on overall quality of life, physical activity, or cognitive function of the donors.^1^, ^4^

**Research in context**

**Evidence before this study**

We searched for randomised trials published in English from database inception to March 1, 2019, investigating the effect of intensive approaches to help whole blood donors keep appointments, or of varying the inter-donation interval. We searched PubMed, Scientific Citation Index Expanded, and Embase using relevant terms: “blood donation intervals”, “blood donation frequency”, “blood supply”, “donor health”, “appointments”, and “reminders”. Regarding trials of approaches to remind donors to keep appointments, we could not identify any previous relevant studies. Regarding trials of varying the inter-donation interval, we identified only the INTERVAL trial, a trial of 45 263 donors that showed that, over a two-year period, inter-donation intervals for whole blood donation can be safely reduced to meet blood shortages. However, longer-term data are needed to inform policy more appropriately.

**Added value of this study**

As probably the first randomised trial of the effects of giving blood donors intensive reminders to help keep their appointments, the present study should provide unique insight into this question. Regarding the longer-term effects of varying the inter-donation interval, the present study extended the original INTERVAL trial beyond its initial 2-year period for up to a further 2-year period, recording a set of comprehensive outcomes relating to blood donation, clinical safety, and biochemistry.

**Implications of all the available evidence**

Our results give policy makers in the UK two additional evidence-based options to meet blood supply needs, that is, the use of frequent reminders to help donors keep appointments and shorter inter-donation intervals than are now standard. Our data also quantify the extent of iron depletion within 4 years of repeated donation, thus informing safety guidelines. Finally, our results suggest a need to review the screening method used in the UK to test individuals' eligibility to donate.

These results suggested that, over a duration of about 2 years, blood collection services could safely use shorter donation intervals to meet shortages, such as during periods of high demand.^5^ However, the INTERVAL trial showed that increased donation frequency resulted in a greater number of deferrals (temporary suspension of donors from giving blood) because of low haemoglobin, lower average haemoglobin and ferritin concentrations, and more self-reported symptoms (more self-reported symptoms were seen especially among men).^1^ Hence, it is important to assess the acceptability and sustainability of varying the frequency of whole blood donation for periods longer than 2 years.

We extended the INTERVAL trial for up to a further 2 years to compare the longer-term effects of donating whole blood using standard inter-donation intervals in the UK with shorter inter-donation intervals used in other countries.^6^, ^7^, ^8^ During the extension study, we also compared the use of more intensive reminders to keep blood donation appointments versus the routine reminders used by the NHSBT blood service in England.

## Methods

### Study design and participants

INTERVAL was a parallel group, pragmatic, randomised trial.^1^, ^2^, ^3^ Full details of the INTERVAL trial have been published previously.^1^, ^2^, ^3^ In brief, eligible donors were aged 18 years or older, fulfilled routine criteria for donation, had an email address and access to the internet to respond to web-based questionnaires, and were willing to be randomly assigned to any of the trial's intervention groups at one of the 25 static donor centres of NHSBT, the sole blood provider to the NHS in England, UK.

In the main trial, men were randomly assigned to 12-week (standard), 10-week, or 8-week inter-donation intervals, and women to 16-week (standard), 14-week, or 12-week intervals. Randomisation of donors to sex-specific intervention groups in the ratio of 1:1:1 was done at the coordinating centre using a minimisation algorithm to ensure key characteristics (age, weight, and numbers of new *vs* existing donors) were balanced across trial groups at baseline. Because of the nature of the intervention, it was not possible to mask participants to their allocated inter-donation interval intervention group. During the main trial, donors were followed up for a period of 2 years after randomisation. Routine NHSBT blood donation procedures, including eligibility screening with the copper sulphate test, were adopted because of the pragmatic trial design.

In the extension study reported here, donors nearing completion of their 2-year participation in the main trial were invited by email to continue donating blood at their allocated inter-donation intervals beyond the 2-year period initially agreed (appendix p 22–24). Participants were assigned to active (ie, more intensive) or routine reminders for donation appointments. The active reminder system (as used in the main trial) consisted of a uniform three-step reminder process of email, text message, and telephone call to encourage donation attendance, with a particular focus on donors missing appointments. The routine reminders followed the standard NHSBT protocol, which was less intense (appendix p 22–24).

Donors aged 20 years or older were eligible immediately after completion of their 2-year participation in the main trial, provided they could contribute at least 6 months of follow-up before the end of the main trial follow-up study period (ie, June 16, 2016). Participants gave electronic informed consent. The National Research Ethics Service approved (11/EE/0538) this study.

### Randomisation and masking

**Figure 1:**
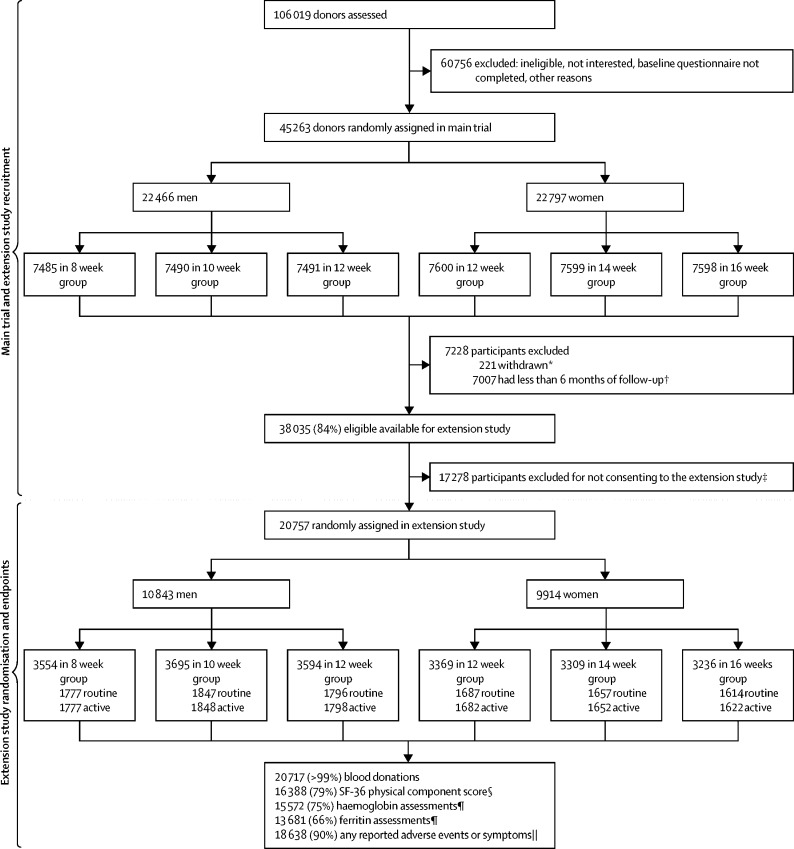
Trial profile CONSORT flowchart showing recruitment, participation, and completeness of main outcomes in the extension study. *Participants who were randomised but later withdrew consent for any further use of their data. †Due to staggered roll-out of the main 2-year trial, only participants expected to attend at least two more sessions were considered eligible for invitation to the extension study. ‡Participants not consenting to the extension study reverted to routine NHS Blood and Transplant reminders (men every 12 weeks, women every 16 weeks). §Number for whom a physical component score could be calculated at the end of the extension trial. ¶Number who provided a research blood sample at the end of the extension trial from which haemoglobin and ferritin were measured. ||Number who responded to at least one question in any of the 6-monthly questionnaires administered during their participation in the extension study.

Participants were block-randomised within each of the main trial groups (inter-donation interval, men: 12, 10, and 8 weeks; women: 16, 14, and 12 weeks) to active (ie, more intensive) or routine reminders for donation appointments (figure 1). Simple 1:1 randomisation was done by the trial's senior data manager (MW) at the coordinating centre using computer-generated random sequences in block sizes of six or eight within the main trial groups. As was the case in the initial trial period, it was not possible to mask participants in the extended study to their allocated inter-donation interval group because of the nature of the intervention. Participants were not informed of their randomly allocated group in the extension study, although individuals returning to routine reminders might have noticed the change. Donors who did not consent to participate in the extension study returned to NHSBT's standard inter-donation intervals (12 weeks for men, 16 weeks for women) and routine appointment reminders. For these participants, consent given at the beginning of the main trial allowed retrieval of anonymised data for blood donations from NHSBT's national database. During the extension study, only the trial's senior data manager (MW) and study coordinator (CM) knew the allocations to active versus routine reminders for purposes of coordination. Laboratory technicians were unaware of the groups to which participants had been randomised.

### Procedures

The extension study used the same procedures as in the main trial.^1^, ^2^, ^3^ These included online administration of 6-monthly questionnaires to monitor donor safety characteristics, and a final questionnaire and collection of a non-fasting research blood sample at the end of the study. These blood samples were transported to a central laboratory for a full blood count analysis (Sysmex XN-2000 haematology analyser, UK BioCentre, Stockport, UK). Ferritin concentrations were measured in stored serum samples with an immunoturbidimetric assay (Roche/Hitachi chemistry analyser, Stichting Huisartsen Laboratorium, Etten-Leur, Netherlands). As with the main trial, at each attendance, donors underwent routine screening for eligibility to donate blood, including pin-prick haemoglobin screening via a gravimetric method (copper sulphate test), followed by the spectrophotometric HemoCue test (HemoCue AB, Ängelholm, Sweden) with venous blood for individuals who did not pass the copper sulphate test (minimum thresholds to donate in England, UK, are 135 g/L for men and 125 g/L for women).^9^

### Outcomes

The primary outcome was the number of whole blood donations made during the extension study expressed as units per year, with standard practice being to donate 1 unit of blood per session (full donation unit 470 mL). The primary outcome was assessed in 20 757 randomly assigned participants, by intention-to-treat. Secondary outcomes related to safety were deferrals of donors (ie, temporary rejection) for low haemoglobin and other factors, haemoglobin and ferritin concentrations, quality of life (using physical and mental wellbeing scores from the Short Form Health Survey, version 2^10^), self-reported symptoms potentially related to blood donation (fainting or feeling faint, tiredness, breathlessness, palpitations, dizziness, chest pain, restless legs, reported low iron concentrations, use of iron supplements, pica), cost-effectiveness of reducing donation intervals (not reported here), and other blood cell-related measures at the end of the extension study reported as secondary exploratory outcomes.

### Statistical analysis

The statistical analysis followed a prespecified plan for the extension study. The sample size calculation was done for the original trial.^1^ Data for men and women were analysed separately by the intention-to-treat principle according to their randomised groups. For prespecified subgroup analyses, ferritin values were log transformed and presented as geometric means and used to classify donors as iron depleted (<15 μg/L) according to WHO criteria.^11^ For all other outcomes, we present means and percentages without adjustment. Analysis of outcomes by active versus routine reminders involved simple differences between groups. For analysis of outcomes by main trial inter-donation interval groups, linear trend was assessed statistically; any non-linearity was identified only graphically. To inform generalisability, we assessed differences in baseline characteristics and outcomes at the end of the main trial, first between individuals who participated in the extension study versus individuals who did not, and second across the main trial randomised inter-donation groups in individuals who took part in the extension study. We compared groups by calculating p values for differences or linear trend using Poisson regression models for rates, normal regression models for continuous outcomes, and logistic regression models for binary outcomes. To minimise potential bias, we adjusted for centre, baseline age, weight, and new donor status, and other covariates when relevant. Precision of estimates were displayed as 95% CIs. For outcomes derived from multiple donation sessions attended, or multiple questionnaires answered by each participant, the 95% CIs were based on robust standard error estimates to avoid optimism in the level precision. Because of the number of statistical tests done, we used the following guidelines for considering whether the results provided strong evidence: p<0·005 for the analyses of whole blood donation rates (ie, the primary outcome), and p<0·0001 for other tests. Analyses were done with Stata, version 13.

There were no protocol amendments or deviations from the trial protocol. This trial is registered with ISRCTN, number ISRCTN24760606, and has completed. An independent data monitoring committee periodically reviewed summaries of the trial data for safety purposes.

### Role of the funding source

The academic investigators and representatives of NHSBT, a funder of the trial, participated in the study design and oversight. The investigators at the trial's academic coordinating centre had sole access to the trial database, and had final responsibility for data collection, data integrity, data analysis and interpretation, as well as manuscript drafting and the decision to submit the manuscript for publication. All authors gave approval to submit for publication.

## Results

Between Oct 19, 2014, and May 3, 2016, of 45 042 participants who completed the main trial, 38 035 (84·4%; 18 754 men, 19 281 women) were invited to participate in the extension study. Of those invited, 20 757 (54·6%; 10 843 men, 9914 women) consented and were randomly assigned to active versus routine appointment reminders (figure 1, appendix p 12). The percentage of participants invited and those consenting were similar across the main trial's sex-specific randomised inter-donation interval groups (figure 1). Median follow-up during the extension study was 1·1 years (IQR 0·7–1·3).

**Figure 2:**
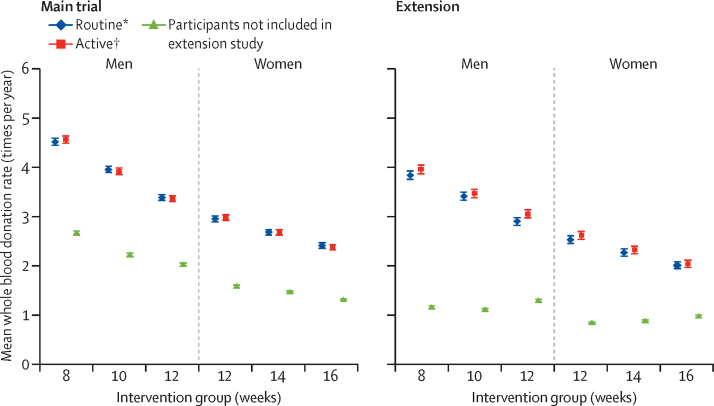
Whole blood donation rate during the main trial and in the extension study by sex and inter-donation intervals All participants in the main trial were allocated to active reminders. Participants not included in the extension study automatically reverted to standard inter-donation intervals (12 weeks for men, 16 weeks for women) at their completion of the main trial, with anonymised lookup of blood donation information from NHS Blood and Transplant records made possible by consent given at the beginning of the main trial. The blood donation rates for these participants during the period of the extension study are shown according to the original randomised groups, purely for comparison purposes, even though they had all reverted to the standard inter-donation intervals. Error bars denote 95% CI. *Allocated to routine reminders in the extension study. †Allocated to active reminders in the extension study.

Participants who consented to the extension study differed from participants who did not in several characteristics recorded at the beginning and during the main trial (appendix pp 3–4). Compared with participants who did not take part, participants were older (by a mean of 7·4 years [95% CI 7·1–7·6]), more committed and adherent within the main trial (donating 79% [95% CI 77–82] more blood), had fewer deferrals, and had a lower frequency of self-reported symptoms (appendix p 3–4). Donation rates in donors who did not take part in the extension study (ie, individuals reverting to standard inter-donation intervals at the end of the main trial) were lower than in individuals who participated (figure 2).

Information on the primary outcome was available for 20 717 (99·8%) of 20 757 participants (figure 1). Baseline characteristics of participants were well balanced across the randomised active versus routine reminders trial groups (appendix p 8). Mean whole blood donation rates for active versus routine reminders in men were 3·50 (95% CI 3·45–3·54) versus 3·39 (3·34–3·44) units per year, or a mean difference of 0·11 (95% CI 0·04–0·17; p=0·00028) units per year (figure 2; appendix pp 9, 13). Corresponding results in women were 2·33 (95% CI 2·30–2·37) versus 2·28 (2·24–2·31) units per year, or mean difference of 0·06 units per year (95% CI 0·01–0·11; p=0·0094). No significant differences were observed between the active and routine reminder groups in outcomes related to safety (appendix p 9). The effect of active reminders on blood donation rates did not vary according to inter-donation intervals (figure 2; interaction test p=0·86 in men and p=0·55 in women).

From the 20 757 participants, availability of secondary outcomes assessed at the end of the extension study varied: 20 717 (99·8%) for deferrals per donation session attended; 18 638 (89·8%) for self-reported symptoms; 16 388 (79·0%) for physical wellbeing score; 15 572 (75·0%) for haemoglobin and other blood cell measures; and 13 681 (65·9%) for ferritin concentration (figure 1). Availability of these outcomes was broadly similar between randomised groups (appendix p 12).

In the participants included in the extension study, the effects of shorter inter-donation intervals during the first 2 years were consistent with the main trial findings, including lower concentrations of haemoglobin and ferritin (appendix p 5). Exploratory analyses showed that shorter inter-donation intervals also led to lower concentrations of other commonly assessed haematological variables at the end of the main trial (appendix p 6). In this subset of participants, however, there was no evidence of the effects of shorter inter-donation intervals on self-reported symptoms (eg, tiredness, feeling faint, dizziness, breathlessness), although there was a higher reported frequency of doctor-diagnosed low iron concentrations and prescription of iron supplements in men (both p<0·0001; appendix p 7).

**Figure 3:**
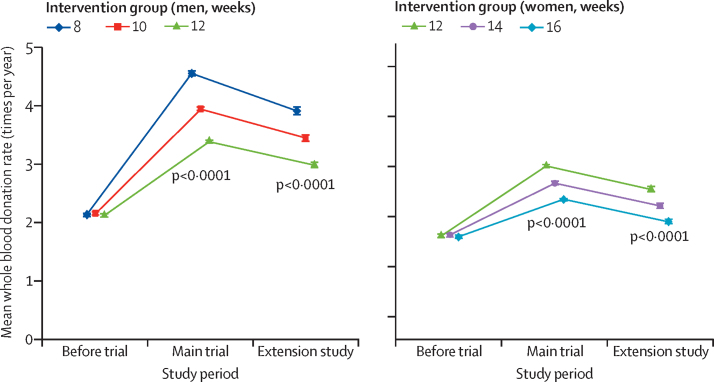
Whole blood donation rates during the extension study, the main trial period, and in the previous 2 years by sex and inter-donation intervals The p values compare across inter-donation intervals and are adjusted for baseline characteristics (centre, age, weight, new donor status). Minimum inter-donation intervals allowed before the trial were 12 weeks for men and 16 weeks for women. Error bars denote 95% CI.

**Figure 4:**
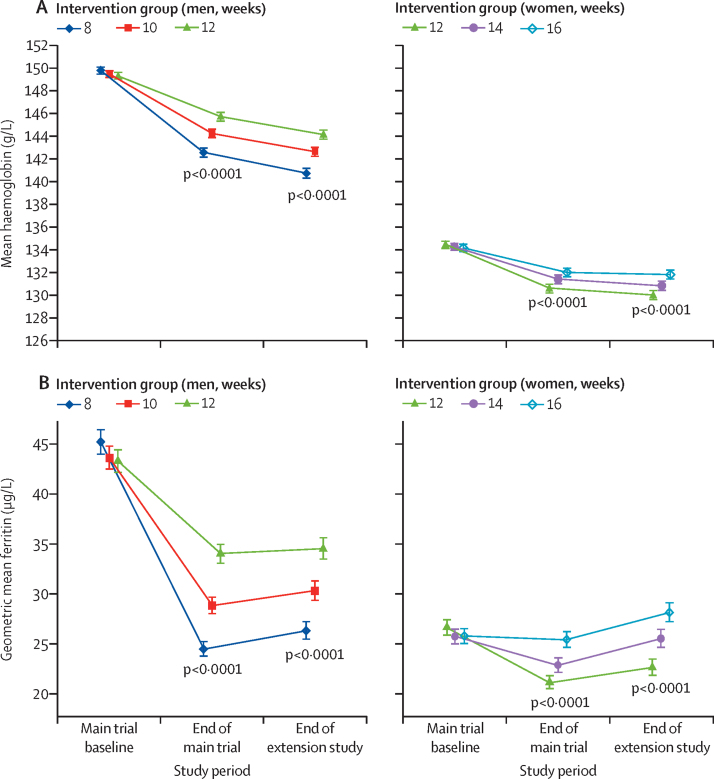
Haemoglobin (A) and ferritin (B) concentrations at the end of the extension study, end of the main trial period, and at baseline by sex and inter-donation intervals Analysis is restricted to participants in the extension study. The p values assess trends across inter-donation intervals, adjusted for baseline characteristics (centre, age, weight, new donor status, and haemoglobin [A] or log_e_ ferritin [B]). Error bars denote 95% CI.

Baseline characteristics of participants were broadly similar across the inter-donation interval groups (appendix p 5). Donors continuing to donate at shorter inter-donation intervals gave more blood during the extension study than individuals continuing on the longer intervals (men an extra 0·23 units per year [95% CI 0·21–0·25], women an extra 0·14 units per year [0·12–0·15], per week shorter interval based on linear trend, both p<0·0001; figure 3, table 1). There were no clear differences across trial groups in physical and mental wellbeing scores or reported frequency of self-reported symptoms other than a higher reported frequency of doctor diagnosed low iron concentrations and prescription of iron supplements in men (table 1; appendix pp 10, 14). Similarly, there were no differences across trial groups in the frequency of serious adverse events (eg, heart failure, myocardial infarction, stroke, falls, or transport accidents; table 2). However, donors allocated to shorter inter-donation intervals had more deferrals for low haemoglobin (odds ratio per week shorter inter-donation interval 1·19 [95% CI 1·15–1·22] in men and 1·10 [1·06–1·14] in women), and had lower mean haemoglobin (difference per week shorter inter-donation interval −0·84 g/L [95% CI −0·99 to −0·70] in men and −0·45 g/L [–0·59 to −0·31] in women) and ferritin concentrations (percentage difference per week shorter inter-donation interval −6·5% [95% CI −7·6 to −5·5] in men and −5·3% [–6·5 to −4·2] in women) at the end of the extension study (all p<0·0001; table 1, figure 4; appendix p 15). Shorter inter-donation intervals also led to lower concentrations of other commonly assessed haematological variables at the end of the extension study (appendix p 11). The proportion of individuals donating blood with haemoglobin concentrations less than the minimum regulatory threshold and individuals with ferritin less than 15 μg/L was higher in donors allocated to shorter intervals than in individuals allocated to the standard donation intervals (appendix p 16).

**Table 1:** Outcomes during the extension study by sex and inter-donation groups

	**Men**				**Women**			
	8 weeks	10 weeks	12 weeks	p value*	12 weeks	14 weeks	16 weeks	p value*
Participants†	3554 (33%)	3695 (34%)	3594 (33%)	··	3369 (34%)	3309 (33%)	3236 (33%)	··
Follow-up time, years (median, IQR)	1·2 (0·8–1·3)	1·2 (0·8–1·3)	1·1 (0·7–1·3)	··	1·1 (0·6–1·3)	1·1 (0·6–1·3)	1·0 (0·6–1·2)	··
Whole blood donation rate (times per year)	3·90 (3·83–3·96)	3·44 (3·38–3·49)	2·97 (2·93–3·02)	<0·0001	2·57 (2·53–2·62)	2·29 (2·25–2·33)	2·02 (1·99–2·06)	<0·0001
Deferral for low haemoglobin‡	5·94% (5·56–6·33)	4·43% (4·07–4·79)	3·04% (2·71–3·38)	<0·0001	6·22% (5·70–6·74)	5·19% (4·68–5·70)	4·42% (3·92–4·93)	<0·0001
Deferral for other reasons‡	3·32% (3·04–3·60)	3·71% (3·39–4·04)	3·76% (3·40–4·12)	0·064	4·25% (3·84–4·67)	5·18% (4·68–5·68)	5·20% (4·66–5·75)	0·003
Fainting at donation session‡	0·15% (0·09–0·21)	0·18% (0·11–0·26)	0·17% (0·09–0·24)	0·61	0·52% (0·37–0·67)	0·45% (0·30–0·60)	0·48% (0·30–0·66)	0·68
SF-36 physical wellbeing score	56·5 (56·3–56·7)	56·6 (56·4–56·7)	56·4 (56·3–56·6)	0·94	56·6 (56·4–56·8)	56·4 (56·2–56·7)	56·3 (56·1–56·5)	0·11
SF-36 mental wellbeing score	54·3 (54·0–54·5)	54·2 (54·0–54·4)	54·1 (53·8–54·3)	0·63	53·3 (53·0–53·6)	53·2 (52·9–53·5)	53·0 (52·7–53·2)	0·077
Haemoglobin (g/L)	140·8 (140·3–141·2)	142·7 (142·3–143·1)	144·2 (143·8–144·6)	<0·0001	130·0 (129·6–130·4)	130·8 (130·4–131·2)	131·8 (131·4–132·2)	<0·0001
Haemoglobin <135 g/L (men) or <125 g/L (women)§	22·29% (20·38–24·19)	16·26% (14·72–17·81)	14·01% (12·58–15·43)	<0·0001	21·80% (19·87–23·73)	18·81% (17·00–20·62)	16·81% (15·14–18·49)	<0·0001
Ferritin (μg/L)¶	26·3 (25·5–27·2)	30·3 (29·4–31·3)	34·5 (33·5–35·6)	<0·0001	22·6 (21·8–23·4)	25·5 (24·7–26·4)	28·2 (27·2–29·1)	<0·0001
Ferritin <15 μg/L§	21·19% (19·20–23·18)	16·41% (14·75–18·08)	11·87% (10·46–13·28)	<0·0001	25·00% (22·85–27·15)	20·04% (18·07–22·01)	18·46% (16·62–20·30)	<0·0001
Serious adverse events‖	2·35% (1·83–2·88)	2·75% (2·19–3·31)	2·88% (2·30–3·45)	0·25	2·73% (2·15–3·32)	3·48% (2·82–4·15)	3·21% (2·57–3·86)	0·30

Data are mean or percentage (95% CI) unless otherwise stated. SF-36=36-item short-form health survey.

* p values are for linear trend across groups, from analyses adjusted for baseline characteristics (centre, age, weight, new donor status) and value of the outcome at baseline (when available).

† Additional missing data during the extension study were: <0·2% for blood donation, deferrals, or fainting; 21·0% for SF-36 Physical/Mental wellbeing scores, 25·0% for haemoglobin, 34·1% for ferritin. Higher SF-36 scores indicate better physical or mental wellbeing (0–100 scale range).

‡ Deferral or fainting rate per donation session attended during the extension study.

§ Among individuals donating blood at end of the extension study.

¶ Values are geometric means.

‖ Percentage of participants reporting any serious adverse events during the extension study, in any of the 6-monthly questionnaires, including doctor-confirmed heart failure, heart attack, angina, stroke, or transient ischaemic attack; and hospital visit for falls or transport accidents.

**Table 2:** Adverse events during the extension study by inter-donation interval groups

		Grade*	**Overall**		**Men (n=10 843 in extension)**			**Women (n=9914 in extension)**		
			N	n (%)	8 weeks	10 weeks	12 weeks	12 weeks	14 weeks	16 weeks
Any self-reported serious adverse events†		··	18 550	536 (2·9%)	75 (2·4%)	91 (2·8%)	93 (2·9%)	82 (2·7%)	102 (3·5%)	93 (3·2%)
	Doctor diagnosed heart problems	3	18 528	69 (0·4%)	14 (0·4%)	20 (0·6%)	17 (0·5%)	4 (0·1%)	5 (0·2%)	9 (0·3%)
	Doctor diagnosed heart failure	3	18 528	18 (0·1%)	5 (0·2%)	4 (0·1%)	5 (0·2%)	1 (0·0%)	1 (0·0%)	2 (0·1%)
	Doctor diagnosed heart attack	3	18 526	20 (0·1%)	7 (0·2%)	5 (0·2%)	4 (0·1%)	1 (0·0%)	0 (0·0%)	3 (0·1%)
	Doctor diagnosed angina	3	18 526	24 (0·1%)	6 (0·2%)	6 (0·2%)	5 (0·2%)	1 (0·0%)	4 (0·1%)	2 (0·1%)
	Doctor diagnosed stroke	3	18 527	17 (0·1%)	4 (0·1%)	7 (0·2%)	1 (0·0%)	2 (0·1%)	1 (0·0%)	2 (0·1%)
	Doctor diagnosed transient ischaemic attack	3	18 527	21 (0·1%)	5 (0·2%)	5 (0·2%)	5 (0·2%)	2 (0·1%)	1 (0·0%)	3 (0·1%)
	Visit to hospital for a fall	3	18 533	337 (1·8%)	39 (1·2%)	39 (1·2%)	47 (1·5%)	64 (2·1%)	81 (2·8%)	67 (2·3%)
	Visit to hospital for transport accident	3	18 516	150 (0·8%)	26 (0·8%)	36 (1·1%)	36 (1·1%)	17 (0·6%)	17 (0·6%)	18 (0·6%)
Any symptom self-reported		1–2	18 554	9732 (52·5%)	1581 (49·6%)	1556 (47·1%)	1476 (45·6%)	1764 (58·8%)	1699 (58·0%)	1656 (57·2%)
	Fainting or feeling faint	1–2	18 534	2085 (11·3%)	325 (10·2%)	302 (9·2%)	269 (8·3%)	424 (14·1%)	390 (13·3%)	375 (13·0%)
	More tired than usual	1–2	18 537	5198 (28·0%)	864 (27·1%)	823 (24·9%)	800 (24·8%)	947 (31·6%)	881 (30·1%)	883 (30·5%)
	Palpitations	1–2	18 502	2217 (12·0%)	271 (8·5%)	286 (8·7%)	261 (8·1%)	498 (16·6%)	435 (14·9%)	466 (16·2%)
	Dizziness	1–2	18 533	3197 (17·3%)	457 (14·4%)	456 (13·8%)	419 (13·0%)	657 (21·9%)	617 (21·1%)	591 (20·4%)
	Restless legs syndrome	1–2	18 464	4158 (22·5%)	642 (20·2%)	642 (19·5%)	613 (19·0%)	776 (26·0%)	753 (25·8%)	732 (25·4%)

Data presented are n (%) unless otherwise stated. Adverse events listed in this table were ascertained only through self-report questionnaires and mapped to Common Terminology Criteria for Adverse Events grading using heuristic criteria. For adverse events of grade 1–2, only those occurring in 10% or more of patients are reported.

* Grading with reference to Common Terminology Criteria for Adverse Events version 5.0.

† Number and percentage of participants reporting any serious adverse events during the extension study in any of the 6-monthly questionnaires, including doctor-confirmed heart failure, heart attack, angina, stroke, or transient ischaemic attack; or hospital visit for falls or transport accidents. Study participants could contribute to more than one outcome in this table.

During the extension study, blood donation rates in each trial group were 14·6% (95% CI 13·1–14·2) lower than during the main trial (figure 3). In comparison with the main trial, frequency of self-reported symptoms and rates of deferral for low haemoglobin increased further (appendix pp 14–15), while mean haemoglobin concentrations decreased further (figure 4A), especially in men. By contrast, mean ferritin concentrations increased somewhat, especially in women (figure 4B). Corresponding changes in other haematological variables (appendix p 17) showed similar results to haemoglobin for some traits (eg, lower mean corpuscular haemoglobin and mean corpuscular haemoglobin concentration) and similar to ferritin for other traits (eg, higher mean haematocrit, mean corpuscular volume, and reticulocyte haemoglobin equivalent). There was no evidence that laboratory machine drift or technical errors, as judged by evaluation of internal quality control samples, could explain differences in the above-mentioned variables.

The proportion of donors who reported that their doctor had prescribed iron supplements increased through the duration of the INTERVAL trial up to 4·0% (95% CI 3·7–4·4) by the end of the extension study, and together with individuals reporting the use of over-the-counter iron supplements, comprised 1396 (16%) of 8594 men and 1732 (22%) of 7803 women by the end of the extension study, with higher proportions in donors allocated to shorter intervals (appendix p 18).

In post-hoc analyses, which stratified comparisons according to patterns of reported use of any iron supplements during the main trial or extension study, the decrease in mean haemoglobin concentrations was larger (appendix p 19) and the increase in mean ferritin concentrations no longer apparent (appendix p 20) among the participants who did not report using iron supplements throughout the trial. Similarly, stratified post-hoc results for reticulocyte haemoglobin concentration (appendix p 21) showed increased concentrations during the extension study even in the subgroup of participants who were iron supplement-naive.

## Discussion

This trial extended the intervention and follow-up periods of INTERVAL, a randomised trial of varying inter-donation intervals in whole blood donors. We also did a randomised comparison of different approaches to remind donors to keep blood donation appointments. This extension study's main result was that, over a period of 2–4 years, shorter inter-donation intervals and more intensive reminders resulted in more blood being collected.

Our trial was notable because it quantified key measures that blood services aim to balance in maintaining the blood supply while safeguarding the health of donors. Our extension study showed that, beyond a 2-year period, each week reduction in time between donations led to an increase of 0·23 units in the amount of blood collected per year in men and of 0·14 units in women compared with the donation intervals currently used in the UK (ie, 12 weeks in men and 16 weeks in women).^6^ With regard to use of more intensive approaches to remind donors of appointments, our study showed a mean increase of 0·11 units of blood per year in men and of 0·06 units of blood per year in women. These modest increases due to additional reminders could potentially translate to collection of an approximate extra 75 000 units of blood from a donor base of 900 000 with about 47% of men and 53% of women (ie, the approximate size of the current donor base in England, UK). If more intensive reminders (eg, a telephone call when an appointment is missed) could be done at little additional cost, then the gain in the amount of blood collected could be worthwhile, at least for priority blood groups.^12^, ^13^, ^14^ The cost implications of a range of alternative policies to encourage blood donation, partly based on the INTERVAL trial, have been published elsewhere.^15^

Regarding safety, the trial showed that reducing inter-donation intervals during the extension study did not have major adverse effects on self-reported mental and physical wellbeing, specific symptoms potentially related to blood donation, or in other major adverse events we recorded. These results extend those from the main trial showing that reducing inter-donation intervals did not result in major adverse events or impaired wellbeing.^1^, ^8^ However, when compared with the initial 2 years of the trial, the proportion of donors reporting specific symptoms increased during the extension study, suggesting a potential cumulative effect over a longer period of time.

Use of shorter donation intervals during the extension study also resulted in changes in biomarkers of iron homoeostasis, resulting in more deferrals for low haemoglobin, decreased mean haemoglobin and serum ferritin concentrations, and changes in other red blood cell parameters suggesting lower iron availability and lower incorporation into red blood cells.^16^ As observed for the main trial, there were modest absolute decreases in mean haemoglobin concentrations and other red blood cell parameters at the end of the extension study. By contrast, proportional reductions were larger for serum ferritin, with up to 21% of men and 25% of women with serum ferritin concentrations less than 15 g/L at the end of the extension study. These results are consistent with previous observational studies, suggesting that shorter inter-donation intervals are associated with sustained and progressively lower iron availability.^17^, ^18^ However, although shorter donation intervals resulted in further decreases in haemoglobin levels in the extension study, serum ferritin concentrations actually increased somewhat (in parallel with increases in the haemoglobin concentration of reticulocytes). Exploratory analyses suggest that this result could be explained by the higher proportion of donors who reported using iron supplements,^19^, ^20^ as by the end of the extension study 16% of men and 22% of women had either been prescribed iron supplements, or reported taking over-the-counter iron supplements.^21^, ^22^

Our findings could have several potential implications for blood donation practice and policy. First, our results provide evidence for the long-term safety of more frequent donation and give policy makers in the UK the option to allow more frequent collection from donors than is now standard.^6^ Nevertheless, total reliance on this strategy might make a blood service overly dependent on a subgroup of donors who are the most resilient to iron depletion, either biologically or through iron supplementation.^23^ Another option would be to use shorter inter-donation intervals only for more resilient donors, if such donors could be identified in advance by demographic, haematological, or genetic characteristics.^1^

Second, our data provide convincing evidence of the cumulative effect on haemoglobin concentrations and iron stores of donating blood frequently, which should inform safety guidelines for blood services that allow more frequent donation than in the UK (eg, USA, France, and Germany). Our results support the recent changes in the Canadian Blood Services that have increased the minimum inter-donation interval in women to reduce iron deficiency and deferrals for low haemoglobin.^24^

Third, given the decrease in haemoglobin concentrations we observed over a longer period, it is essential for blood services to protect the health of donors by adopting appropriate screening methods to test donors' eligibility to donate whole blood.^25^ To evaluate the relative merits of different screening methods in the context of NHSBT, the COMPARE study (ISRCTN90871183) aims to provide a systematic, within-person comparison of different methods to measure haemoglobin concentrations in whole blood donors to inform approaches for routine eligibility checks in England, UK. Furthermore, other blood services have implemented or are evaluating additional approaches to detect iron deficiency, such as ferritin monitoring in selected blood donors.^22^, ^26^

Fourth, our findings underscore the potential benefits of effective communication with blood donors to encourage attendance, especially in an appointments-based system such as used by NHSBT in England, UK.

Our study had strengths. Because we evaluated the long-term safety and efficiency of frequent donation beyond a 2-year period in a randomised study, our trial provides more reliable insights than do observational studies, which are susceptible to confounding. The trial recorded a comprehensive set of outcomes relating to blood donation, clinical safety, and biochemistry, and provided almost complete outcome data for amount of blood collected and deferrals because of low haemoglobin.

The study also had limitations. Continuation into the extension study was accepted by 55% of those invited, and therefore analyses are less powerful than in the main trial. Although the participants in the main trial were broadly representative of the national donor population in England, UK, individuals in the extension study were an older and more committed subset of blood donors; they had also had fewer deferrals for low haemoglobin and reported fewer symptoms. Hence, caution is needed in extrapolating the findings to the general population of blood donors. For example, more intensive reminders could yield even more blood donations in less selected groups than our enthusiastic donors who decided to enrol in the extension study (who tend to miss few opportunities to give blood anyway).^27^, ^28^

During the extension study, half of the participants were switched from active to routine reminders, a switch which could explain a small part of why blood donation during the extension study decreased by about 15% compared with the initial 2 years of the trial. However, drivers of the decreased donation rate between the main trial and the extension study could not be established given the study design.^29^ Although participants were not informed of their randomly allocated group in the extension study, individuals returning to routine reminders might have noticed the change and potentially be influenced by the active reminders from the main trial.

The study relied on self-reported information for some outcomes (eg, symptoms), which might be susceptible to reporting biases and incompleteness (ie, missing data). We did not have accurate information from the 6-monthly questionnaires about the timing of reported iron supplement use, and therefore could not distinguish whether it might be related to previous deferral or subsequent donation.

In summary, during a period of 2–4 years, collection of substantially more blood without a detectable effect on donors' mental and physical wellbeing was achieved through more frequent donation than is standard practice in the UK and more intensive reminders to keep blood donation appointments. However, compared with the initial 2 years of the trial, extension of this approach resulted in further lowering of haemoglobin concentrations, more deferrals, and higher rates of self-reported symptoms.

## Data sharing

The INTERVAL Study Group has previously published its trial protocol, statistical analysis plan, informed consent form, and other relevant study documents. Bona fide scientists can seek access to relevant de-identified individual participant data (and a copy of the trial's data dictionary) by applying to the INTERVAL Data Access Committee after print publication of the current manuscript at the following: helpdesk@intervalstudy.org.uk. The INTERVAL Data Access Committee review (supplemented, when required, by expertise from additional scientists external to the Committee) applications according to usual academic criteria of scientific validity and feasibility. Following approval by the INTERVAL Data Access Committee, a material transfer or research collaboration agreement will be agreed and signed with the applicants. Applicants might be requested to provide reimbursement of data management or preparation costs, as the INTERVAL trial is no longer in receipt of funding. Applicants will be required to provide updates to the INTERVAL Data Access Committee on their use of the INTERVAL trial data, including provision of copies of any publications. Applicants will be required to adhere in publications with the INTERVAL trial's policy for acknowledgment of the trial's funders, stakeholders, and scientific or technical contributors.
