# Supplementary material for: Longer-term efficiency and safety of increasing the frequency of whole blood donation (INTERVAL): extension study of a randomised trial of 20 757 blood donors
Source: Lancet Haematol. 2019 Aug 2;6(10):e510–20. doi: 10.1016/S2352-3026(19)30106-1 (PMC7029279; doi:10.1016/S2352-3026(19)30106-1)
Supplement: Supplementary appendix [file mmc1.pdf]

THE LANCET
Haematology

Supplementary appendix

This appendix formed part of the original submission and has been peer reviewed. We post it as supplied by the authors.

Supplement to: Kaptoge S, Angelantonio ED, Moore C, et al. Longer-term efficiency and safety of increasing the frequency of whole blood donation (INTERVAL): extension study of a randomised trial of 20 757 blood donors. *Lancet Haematol* 2019; published online Aug 2. http://dx.doi.org/10.1016/S2352-3026(19)30106-1.

Supplementary Material

Longer-term safety and efficiency of varying the frequency of whole blood donation (INTERVAL): extension of a randomised trial in 20,757 blood donors

eTable 1. Baseline characteristics by sex among those invited in the extension study. 3
eTable 2. Symptoms and other haematological markers during main trial by sex among those invited in the extension study. 4
eTable 3. Baseline characteristics by sex and inter-donation intervals among those in the extension study.. 5
eTable 4. Haematological markers at end of main trial by sex and inter-donation intervals among those in the extension study.. 6
eTable 5. Reported symptoms during the main trial by sex and inter-donation intervals among those in the extension study. 7
eTable 6. Baseline characteristics by sex and reminder approaches in the extension study. ... 8
eTable 7. Blood donations and other outcomes during the extension study by and reminder approaches.. 9
eTable 8. Reported symptoms during the extension study by inter-donation intervals......... 10
eTable 9. Haematological markers at end of extension study by inter-donation intervals..... 11

eFigure 1. Extended CONSORT flow chart: recruitment, participation, and completeness of main outcomes in the extension study. 12
eFigure 2. Whole blood donation rates during the extension study by sex and reminder approaches... 13
eFigure 3. Reported symptoms by sex and inter-donation intervals among participants in the extension study.. 14
eFigure 4. Low haemoglobin deferral rates by sex and inter-donation intervals among participants in the extension study. 15
eFigure 5. Haemoglobin concentrations below regulatory thresholds (a), and ferritin concentrations <15 µg/L (b), by sex and inter-donation intervals at the end of the extension study, end of the main trial period, and at baseline. 16
eFigure 6. Trends in standardized haematological characteristics by inter-donation intervals restricted to participants with ferritin measured at the end of the extension study.............. 17
eFigure 7. Trends in reported use of iron supplements by inter-donation intervals among participants in the extension study. 18
eFigure 8. Haemoglobin concentrations by inter-donation intervals, stratified according to reported use of any iron supplements during the main trial or during the extension study, among participants in the extension study.. 19
eFigure 9. Ferritin concentrations by inter-donation intervals, stratified according to reported use of any iron supplements during the main trial or during the extension study among participants in the extension study. 20
eFigure 10. Reticulocyte haemoglobin concentrations by inter-donation intervals, stratified according to reported use of any iron supplements during the main trial or during the extension study among participants in the extension study... 21

eAppendix. Extension study invitations, contributing data, and protocols for appointment reminders..22

eTable 1. Baseline characteristics by sex among those invited in the extension study.

	Men (N = 18754 invited)			Women (N = 19281 invited)		
Characteristics	**In extension**	**Not in extension**	**P-value[6]**	**In extension**	**Not in extension**	**P-value[6]**
No (% out of total invited)[1]	10843 (58%)	7911 (42%)		9914 (51%)	9367 (49%)	
**At beginning of main trial**						
Age (years)	48.1 (13.2)	41.5 (14.2)	<0.0001	45.2 (13.4)	37.6 (13.4)	<0.0001
Weight (kg)	85.3 (14.2)	84.7 (14.9)	0.008	71.6 (14.4)	71.8 (15.2)	0.458
New donor[2]	497 (4.6%)	888 (11.2%)	<0.0001	689 (6.9%)	1236 (13.2%)	<0.0001
Blood donation rate in previous 2 years (times/yr)	2.17 (0.87)	1.94 (0.95)	<0.0001	1.82 (0.82)	1.61 (0.87)	<0.0001
Deferral for low Hb per attendance in previous 2 years (%)[3]	490 (1.03%)	305 (1.08%)	0.548	1255 (3.34%)	1121 (3.90%)	0.001
SF-36 Physical wellbeing score	56.8 (4.3)	56.9 (4.8)	0.715	57.1 (4.4)	57.0 (4.9)	0.365
SF-36 Mental wellbeing score[7]	55.1 (5.5)	53.9 (6.6)	<0.0001	54.0 (6.1)	53.0 (7.0)	<0.0001
Haemoglobin (g/L)	149.5 (9.7)	149.7 (10.1)	0.138	134.3 (8.9)	133.5 (9.5)	<0.0001
Haemoglobin <135 g/L (men) or <125 g/L (women) (%)[4]	326 (3.9%)	171 (5.2%)	0.005	769 (10.7%)	430 (13.0%)	0.001
Ferritin (µg/L)[5]	44.0 (27.0-73.0)	45.6 (28.0-80.0)	0.007	26.0 (16.0-45.0)	23.2 (13.0-42.0)	<0.0001
Ferritin <15 µg/L (%)[4]	538 (6.8%)	300 (9.5%)	<0.0001	1367 (19.9%)	828 (26.2%)	<0.0001
**During main trial**						
Blood donation rate (times/yr)	3.95 (1.10)	2.27 (1.57)	<0.0001	2.68 (0.81)	1.43 (1.10)	<0.0001
Deferral for low Hb per attendance (%)[3]	3380 (3.62%)	2138 (5.25%)	<0.0001	3333 (5.49%)	2764 (8.41%)	<0.0001
Deferral for other reasons per attendance (%)[3]	3536 (3.79%)	2355 (5.78%)	<0.0001	3371 (5.56%)	2858 (8.69%)	<0.0001
Fainting at donation session per attendance (%)[3]	204 (0.22%)	179 (0.44%)	<0.0001	362 (0.60%)	318 (0.97%)	<0.0001
**At end of main trial (ie, beginning of the extension study)**						
SF-36 Physical wellbeing score[7]	56.7 (4.8)	56.1 (5.7)	<0.0001	56.7 (5.2)	55.8 (6.3)	<0.0001
SF-36 Mental wellbeing score[7]	54.3 (6.4)	52.9 (7.6)	<0.0001	53.1 (7.2)	51.6 (8.2)	<0.0001
Haemoglobin (g/L)	144.2 (11.6)	146.0 (12.1)	<0.0001	131.3 (11.2)	131.8 (11.7)	0.035
Haemoglobin <135 g/L (men) or <125 g/L (women) (%)[4]	1156 (13.7%)	363 (10.9%)	<0.0001	1246 (17.0%)	572 (17.1%)	0.974
Ferritin (µg/L)[5]	28.9 (17.0-52.0)	36.7 (20.0-69.0)	<0.0001	23.0 (13.0-43.0)	24.7 (14.0-49.0)	<0.0001
Ferritin <15 µg/L (%)[4]	1354 (17.5%)	455 (14.6%)	<0.0001	1664 (24.7%)	721 (23.1%)	0.082
Completed questionnaire	10287 (94.9%)	3552 (44.9%)	<0.0001	9322 (94.0%)	3913 (41.8%)	<0.0001
Gave blood sample	10605 (97.8%)	4049 (51.2%)	<0.0001	9614 (97.0%)	4350 (46.4%)	<0.0001

Data presented are mean (standard deviation) or number of participants (%) unless otherwise stated.
[1] Additional missing data: None for age, weight, or donation history; SF-36 Physical/Mental wellbeing scores (0.7% baseline, 29.8% end of main trial), haemoglobin (1.9% baseline, 24.8% end of main trial), ferritin (6.4% baseline, 30.6% end of main trial).
[2] A participant who has not previously provided a full blood donation.
[3] Total number (%) of deferrals or faints out of all attendances within 2-year period.
[4] Amongst those donating blood at baseline or end of main trial.
[5] Values are geometric means and interquartile ranges.
[6] P-values are for group differences, from unadjusted analyses.

eTable 2. Symptoms and other haematological markers during main trial by sex among those invited in the extension study.

	Men (N = 18754 invited)			Women (N = 19281 invited)		
Characteristics	**In extension**	**Not in extension**	**P-value[2]**	**In extension**	**Not in extension**	**P-value[2]**
No (% out of total invited)[1]	10843 (58%)	7911 (42%)		9914 (51%)	9367 (49%)	
**Reported 6-monthly during main trial**						
Fainting or feeling faint (%)	3.71 (3.48, 3.95)	6.67 (6.19, 7.16)	<0.0001	6.87 (6.54, 7.21)	11.50 (10.92, 12.07)	<0.0001
More tired than usual (%)	14.49 (14.01, 14.96)	21.81 (20.97, 22.64)	<0.0001	19.08 (18.53, 19.64)	28.28 (27.46, 29.11)	<0.0001
More breathless than usual (%)	4.39 (4.13, 4.66)	8.02 (7.48, 8.56)	<0.0001	5.68 (5.37, 5.99)	8.79 (8.29, 9.29)	<0.0001
Palpitations (%)	3.95 (3.69, 4.22)	6.44 (5.93, 6.94)	<0.0001	7.93 (7.54, 8.33)	11.34 (10.73, 11.95)	<0.0001
Dizziness (%)	5.72 (5.42, 6.02)	8.67 (8.12, 9.22)	<0.0001	10.48 (10.05, 10.91)	15.32 (14.66, 15.98)	<0.0001
Chest pain (%)	2.06 (1.89, 2.23)	3.84 (3.47, 4.21)	<0.0001	2.14 (1.94, 2.33)	3.48 (3.16, 3.81)	<0.0001
Restless legs (%)	11.19 (10.71, 11.66)	13.37 (12.66, 14.08)	<0.0001	17.15 (16.54, 17.77)	17.96 (17.21, 18.71)	0.105
Doctor diagnosed low iron levels (%)	2.91 (2.70, 3.11)	6.53 (6.05, 7.00)	<0.0001	4.12 (3.87, 4.37)	8.30 (7.81, 8.78)	<0.0001
Doctor prescribed iron supplements (%)	1.50 (1.35, 1.65)	3.73 (3.37, 4.09)	<0.0001	2.71 (2.50, 2.92)	5.77 (5.36, 6.18)	<0.0001
**Reported over 2 years during main trial**						
Any iron supplements use (%)	13.26 (12.61, 13.92)	17.13 (15.91, 18.35)	<0.0001	19.73 (18.92, 20.53)	23.15 (21.84, 24.47)	<0.0001
Pica (%)	0.65 (0.50, 0.81)	1.53 (1.12, 1.95)	<0.0001	1.61 (1.35, 1.86)	1.87 (1.43, 2.30)	0.310
**At end of main trial (ie, beginning of the extension study)**						
Red blood cell count, RBC ($x10^{12}$/L)	5.02 (0.41)	5.04 (0.42)	0.004	4.58 (0.40)	4.58 (0.40)	0.902
Haematocrit, HCT (%)	45.4 (3.4)	45.7 (3.5)	<0.0001	42.0 (3.4)	42.1 (3.5)	0.658
Mean corpuscular volume , MCV (fL)	90.6 (5.3)	90.8 (5.5)	0.086	91.9 (5.4)	91.9 (5.8)	0.998
Mean corpuscular haemoglobin, MCH (pg)	28.8 (2.2)	29.0 (2.2)	<0.0001	28.8 (2.3)	28.8 (2.3)	0.038
Mean corpuscular Hb concentration, MCHC (g/dL)	31.8 (1.3)	31.9 (1.3)	<0.0001	31.3 (1.4)	31.3 (1.2)	0.238
Reticulocyte Hb equivalent, RET-He (pg)	31.9 (2.3)	32.0 (2.4)	0.018	31.6 (2.3)	31.5 (2.5)	0.009
Platelet count, PLT ($x10^9$/L)	235 (54)	238 (52)	0.003	270 (61)	272 (62)	0.014
White blood cell count, WBC ($x10^9$/L)	6.06 (1.48)	6.18 (1.55)	<0.0001	6.59 (2.74)	6.85 (1.77)	<0.0001
Neutrophil count, NEUT# ($x10^9$/L)	3.47 (1.13)	3.57 (1.20)	<0.0001	3.81 (2.26)	4.01 (1.38)	<0.0001

Data presented are percentage (95% CI) reported over all 6-monthly questionnaires for symptoms (over 2-years for pica), and mean (standard deviation) for haematological markers.

[1] Additional missing data: 11.0% for symptoms (29.8 for any iron supplement use, 30.6% for pica), and 24.8% for haematological markers.

[2] P-values are for group differences, from unadjusted analyses.

eTable 3. Baseline characteristics by sex and inter-donation intervals among those in the extension study.

	Men (N = 10843 in extension)				Women (N = 9914 in extension)			
Characteristics	**8 weeks**	**10 weeks**	**12 weeks**	**P-value[6]**	**12 weeks**	**14 weeks**	**16 weeks**	**P-value[6]**
No (% out of total in extension)[1]	3554 (33%)	3695 (34%)	3594 (33%)	-	3369 (34%)	3309 (33%)	3236 (33%)	-
**At beginning of main trial**								
Age (years)	47.6 (13.3)	48.3 (13.3)	48.5 (13.1)	0.002	45.1 (13.5)	45.3 (13.4)	45.4 (13.4)	0.252
Weight (kg)	85.6 (14.2)	85.3 (14.3)	84.9 (13.9)	0.023	71.6 (14.1)	71.6 (14.6)	71.6 (14.5)	0.896
New donor[2]	170 (4.8%)	171 (4.6%)	156 (4.3%)	0.370	231 (6.9%)	216 (6.5%)	242 (7.5%)	0.334
Blood donation rate in previous 2 years (times/yr)	2.17 (0.88)	2.19 (0.86)	2.16 (0.86)	0.548	1.82 (0.82)	1.83 (0.81)	1.81 (0.82)	0.408
Deferral for low Hb in previous 2 years (%)[3]	157 (1.01%)	170 (1.04%)	163 (1.03%)	0.857	425 (3.3%)	404 (3.21%)	426 (3.53%)	0.389
SF-36 Physical wellbeing score	56.8 (4.4)	56.9 (4.2)	56.8 (4.3)	0.702	57.1 (4.4)	57.0 (4.5)	57.1 (4.4)	0.583
SF-36 Mental wellbeing score	55.2 (5.4)	55.1 (5.5)	55.0 (5.6)	0.079	54.1 (6.2)	54.0 (6.2)	54.1 (6.0)	0.982
Haemoglobin (g/L)	149.8 (9.8)	149.4 (9.7)	149.3 (9.5)	0.042	134.5 (9.0)	134.3 (8.6)	134.2 (9.0)	0.194
Haemoglobin <135 g/L (men) or <125 g/L (women) (%)[4]	96 (3.7%)	117 (4.1%)	113 (3.9%)	0.746	232 (9.7%)	267 (11.1%)	270 (11.2%)	0.107
Ferritin (µg/L)[5]	45.2 (27.3-75.0)	43.6 (27.0-73.0)	43.3 (27.0-71.0)	0.024	26.6 (16.0-47.0)	25.7 (16.0-45.0)	25.8 (16.0-45.0)	0.113
Ferritin <15 µg/L (%)[4]	155 (6.3%)	198 (7.3%)	185 (6.7%)	0.539	419 (18.5%)	476 (20.7%)	472 (20.5%)	0.084
**During main trial**								
Blood donation rate (times/yr)	4.54 (1.24)	3.94 (0.94)	3.38 (0.72)	<0.0001	2.97 (0.91)	2.68 (0.77)	2.40 (0.62)	<0.0001
Deferral for low Hb per attendance (%)[3]	1814 (5.08%)	974 (3.09%)	592 (2.27%)	<0.0001	1501 (6.53%)	1094 (5.42%)	738 (4.22%)	<0.0001
Deferral for other reasons per attendance (%)[3]	1339 (3.75%)	1190 (3.77%)	1007 (3.86%)	0.513	1219 (5.3%)	1138 (5.63%)	1014 (5.8%)	0.032
Fainting at donation session per attendance (%)[3]	67 (0.19%)	68 (0.22%)	69 (0.26%)	0.099	122 (0.53%)	113 (0.56%)	127 (0.73%)	0.033
**At end of main trial (ie, beginning of the extension study)**								
SF-36 Physical wellbeing score	56.8 (4.9)	56.8 (4.7)	56.7 (4.9)	0.351	56.8 (5.1)	56.7 (5.2)	56.6 (5.4)	0.092
SF-36 Mental wellbeing score	54.4 (6.5)	54.3 (6.3)	54.2 (6.6)	0.212	53.0 (7.2)	53.1 (7.1)	53.0 (7.3)	0.910
Haemoglobin (g/L)	14.26 (1.18)	14.43 (1.13)	14.57 (1.13)	<0.0001	13.06 (1.13)	13.14 (1.13)	13.20 (1.11)	<0.0001
Haemoglobin <135 g/L (men) or <125 g/L (women) (%)[4]	452 (17.4%)	391 (13.6%)	313 (10.7%)	<0.0001	460 (19.0%)	430 (17.6%)	356 (14.5%)	<0.0001
Ferritin (µg/L)[5]	24.5 (14.0-43.0)	28.8 (17.0-51.0)	34.0 (21.0-59.0)	<0.0001	21.1 (12.0-39.0)	22.9 (13.0-41.0)	25.4 (14.0-47.0)	<0.0001
Ferritin <15 µg/L (%)[4]	564 (23.6%)	467 (17.7%)	323 (11.9%)	<0.0001	597 (26.8%)	577 (25.6%)	490 (21.8%)	<0.0001
Completed questionnaire	3373 (94.9%)	3503 (94.8%)	3411 (94.9%)	0.998	3155 (93.6%)	3128 (94.5%)	3039 (93.9%)	0.645
Gave blood sample	3463 (97.4%)	3608 (97.6%)	3519 (97.9%)	0.270	3260 (96.8%)	3199 (96.7%)	3142 (97.1%)	0.483

Data presented are mean (standard deviation) or number of participants (%) unless otherwise stated.
[1] Additional missing data: None for age, weight, or donation history; SF-36 Physical/Mental wellbeing scores (0.4% baseline, 6.4% end of main trial), haemoglobin (1.8% baseline, 2.6% end of main trial), ferritin (6.4% baseline, 10.5% end of main trial).
[2] A participant who has not previously provided a full blood donation.
[3] Total number (%) of deferrals or faints out of all attendances within 2-year period.
[4] Amongst those donating blood at baseline or end of main trial.
[5] Values are geometric means and interquartile ranges
[6] P-values are for linear trend across groups, from unadjusted analyses.

eTable 4. Haematological markers at end of main trial by sex and inter-donation intervals among those in the extension study.

	**Men (N = 10843 in extension)**				**Women (N = 9914 in extension)**			
Outcomes	**8 weeks**	**10 weeks**	**12 weeks**	**P-value[2]**	**12 weeks**	**14 weeks**	**16 weeks**	**P-value[2]**
No (% out of total in extension)[1]	3554 (33%)	3695 (34%)	3594 (33%)	-	3369 (34%)	3309 (33%)	3236 (33%)	-
Red blood cell count, RBC ($x10^{12}$/L)	5.03 (5.02, 5.04)	5.03 (5.01, 5.04)	5.01 (5.00, 5.03)	0.842	4.57 (4.55, 4.58)	4.58 (4.57, 4.60)	4.59 (4.58, 4.60)	0.015
Haematocrit, HCT (%)	45.1 (45.0, 45.2)	45.4 (45.3, 45.5)	45.7 (45.6, 45.8)	<0.0001	41.8 (41.7, 42.0)	42.1 (41.9, 42.2)	42.2 (42.1, 42.3)	<0.0001
Mean corpuscular volume , MCV (fL)	89.9 (89.7, 90.1)	90.6 (90.4, 90.8)	91.3 (91.2, 91.5)	<0.0001	91.8 (91.6, 92.0)	91.9 (91.7, 92.1)	92.0 (91.9, 92.2)	0.005
Mean corpuscular haemoglobin, MCH (pg)	28.4 (28.4, 28.5)	28.8 (28.7, 28.9)	29.2 (29.1, 29.2)	<0.0001	28.7 (28.6, 28.8)	28.8 (28.7, 28.8)	28.9 (28.8, 28.9)	<0.0001
Mean corpuscular Hb concentration, MCHC (g/dL)	31.6 (31.6, 31.7)	31.8 (31.7, 31.8)	31.9 (31.9, 32.0)	<0.0001	31.3 (31.2, 31.3)	31.3 (31.3, 31.3)	31.4 (31.3, 31.4)	0.001
Reticulocyte Hb equivalent, RET-He (pg)	31.5 (31.4, 31.6)	31.9 (31.8, 32.0)	32.3 (32.2, 32.3)	<0.0001	31.5 (31.4, 31.6)	31.6 (31.5, 31.7)	31.7 (31.6, 31.8)	<0.0001
Platelet count, PLT ($x10^9$/L)	237 (235, 239)	236 (234, 238)	233 (231, 235)	<0.0001	270 (268, 272)	269 (267, 271)	269 (267, 271)	0.002
White blood cell count, WBC ($x10^9$/L)	6.09 (6.05, 6.14)	6.06 (6.02, 6.11)	6.03 (5.99, 6.08)	0.778	6.56 (6.50, 6.62)	6.55 (6.49, 6.61)	6.65 (6.51, 6.80)	0.185
Neutrophil count, NEUT# ($x10^9$/L)	3.49 (3.45, 3.52)	3.48 (3.44, 3.51)	3.45 (3.41, 3.48)	0.805	3.80 (3.75, 3.84)	3.79 (3.75, 3.83)	3.85 (3.73, 3.97)	0.293

Data presented are mean or percentage (95% CI) unless otherwise stated.
[1] Additional missing data at the end of main trial were 2.7% for each of the haematological markers.
[2] P-values are for linear trend across groups, from analyses adjusted for baseline characteristics (centre, age, weight, new donor status) and value of the outcome at baseline (where available).

eTable 5. Reported symptoms during the main trial by sex and inter-donation intervals among those in the extension study.

	Men (N = 10843 in extension)				Women (N = 9914 in extension)			
Outcomes	**8 weeks**	**10 weeks**	**12 weeks**	**P-value[3]**	**12 weeks**	**14 weeks**	**16 weeks**	**P-value[3]**
No (% out of total in extension)[1]	3554 (33%)	3695 (34%)	3594 (33%)	-	3369 (34%)	3309 (33%)	3236 (33%)	-
**Reported 6-monthly[2]**								
Fainting or feeling faint (%)	4.15 (3.71, 4.59)	3.54 (3.15, 3.93)	3.58 (3.17, 3.98)	0.063	7.01 (6.44, 7.59)	6.95 (6.36, 7.54)	6.84 (6.24, 7.44)	0.732
More tired than usual (%)	15.96 (15.09, 16.83)	13.65 (12.87, 14.44)	14.09 (13.27, 14.90)	0.003	20.49 (19.51, 21.47)	18.09 (17.16, 19.03)	18.94 (17.98, 19.91)	0.031
More breathless than usual (%)	5.07 (4.58, 5.56)	4.03 (3.61, 4.46)	4.19 (3.74, 4.64)	0.011	5.97 (5.42, 6.51)	5.46 (4.95, 5.98)	5.71 (5.17, 6.26)	0.475
Palpitations (%)	4.27 (3.79, 4.75)	4.07 (3.60, 4.55)	3.62 (3.19, 4.06)	0.075	8.30 (7.60, 9.00)	7.35 (6.70, 8.00)	8.43 (7.71, 9.15)	0.735
Dizziness (%)	6.34 (5.77, 6.90)	5.50 (5.00, 6.00)	5.53 (5.02, 6.04)	0.032	10.63 (9.90, 11.36)	10.50 (9.75, 11.25)	10.70 (9.92, 11.48)	0.835
Chest pain (%)	2.29 (1.97, 2.61)	2.00 (1.71, 2.30)	1.95 (1.66, 2.24)	0.166	2.01 (1.70, 2.33)	2.24 (1.90, 2.58)	2.24 (1.88, 2.60)	0.307
Restless legs (%)	11.86 (11.01, 12.70)	11.35 (10.52, 12.17)	10.67 (9.86, 11.48)	0.025	17.47 (16.40, 18.53)	17.01 (15.94, 18.07)	17.58 (16.50, 18.67)	0.964
Doctor diagnosed low iron levels (%)	4.31 (3.88, 4.75)	2.62 (2.28, 2.95)	1.96 (1.66, 2.26)	<0.0001	4.49 (4.04, 4.95)	4.35 (3.90, 4.81)	3.72 (3.30, 4.14)	0.015
Doctor prescribed iron supplements (%)	2.08 (1.76, 2.39)	1.38 (1.13, 1.63)	1.15 (0.92, 1.38)	<0.0001	2.82 (2.45, 3.19)	3.02 (2.63, 3.41)	2.48 (2.13, 2.84)	0.226
**Reported over 2 years**								
Any iron supplements (%)	15.08 (13.88, 16.29)	13.00 (11.89, 14.12)	11.71 (10.64, 12.79)	<0.0001	20.65 (19.24, 22.06)	19.07 (17.70, 20.45)	19.40 (17.99, 20.81)	0.231
Pica (%)	0.84 (0.53, 1.16)	0.46 (0.24, 0.69)	0.66 (0.38, 0.93)	0.520	1.71 (1.25, 2.17)	1.72 (1.26, 2.19)	1.38 (0.96, 1.79)	0.386

Data presented are percentage (95% CI) unless otherwise stated.
[1] Additional missing data during the main trial were 0.6% for each symptom, 6.4% for any iron supplements and 7.2% for pica.
[2] Percentage of individuals reporting these symptoms at 6-monthly intervals, combining data from all the 6-monthly questionnaires during the main trial.
[3] P-values are for linear trend across groups, from analyses adjusted for baseline characteristics (centre, age, weight, new donor status).

eTable 6. Baseline characteristics by sex and reminder approaches in the extension study.

	Men (N = 10843 randomised)		Women (N = 9914 randomised)	
Characteristics	**Routine**	**Active**	**Routine**	**Active**
No (% out of total randomised)[1]	5420 (50%)	5423 (50%)	4958 (50%)	4956 (50%)
**At beginning of main trial**				
Age (years)	48.2 (13.2)	48.0 (13.3)	45.3 (13.6)	45.2 (13.3)
Weight (kg)	85.5 (14.1)	85.1 (14.2)	71.5 (14.2)	71.8 (14.6)
New donor[2]	239 (4.4%)	258 (4.8%)	352 (7.1%)	337 (6.8%)
Blood donation rate in previous 2 years (times/yr)	2.16 (0.86)	2.18 (0.87)	1.82 (0.82)	1.82 (0.81)
Deferral for low Hb per attendance in previous 2 years (%)[3]	243 (1.02%)	247 (1.04%)	610 (3.25%)	645 (3.44%)
SF-36 Physical wellbeing score	56.8 (4.4)	56.9 (4.2)	57.0 (4.5)	57.1 (4.4)
SF-36 Mental wellbeing score	55.2 (5.5)	55.1 (5.5)	54.2 (5.9)	53.9 (6.3)
Haemoglobin (g/L)	149.4 (9.7)	149.6 (9.7)	134.2 (8.7)	134.5 (9.0)
Haemoglobin <135 g/L (men) or <125 g/L (women) (%)[4]	156 (3.7%)	170 (4.1%)	387 (10.7%)	382 (10.6%)
Ferritin (µg/L)[5]	44.4 (28.0-73.0)	43.7 (27.0-73.0)	26.3 (16.0-46.0)	25.8 (16.0-45.0)
Ferritin <15 µg/L (%)[4]	262 (6.6%)	276 (7.0%)	664 (19.3%)	703 (20.5%)
**During main trial**				
Blood donation rate (times/yr)	3.95 (1.09)	3.94 (1.10)	2.69 (0.81)	2.68 (0.82)
Deferral for low Hb per attendance (%)[3]	1652 (3.54%)	1728 (3.70%)	1670 (5.50%)	1663 (5.49%)
Deferral for other reasons per attendance (%)[3]	1771 (3.79%)	1765 (3.78%)	1687 (5.56%)	1684 (5.56%)
Fainting at donation session per attendance (%)[3]	107 (0.23%)	97 (0.21%)	171 (0.56%)	191 (0.63%)
**At end of main trial (ie, beginning of the extension study)**				
SF-36 Physical wellbeing score	56.7 (4.8)	56.7 (4.8)	56.7 (5.1)	56.7 (5.3)
SF-36 Mental wellbeing score	54.3 (6.4)	54.2 (6.4)	53.1 (7.1)	53.0 (7.3)
Haemoglobin (g/L)	144.1 (11.5)	144.3 (11.6)	131.3 (11.2)	131.3 (11.3)
Haemoglobin <135 g/L (men) or <125 g/L (women) (%)[4]	597 (14.2%)	559 (13.3%)	633 (17.3%)	613 (16.8%)
Ferritin (µg/L)[5]	29.2 (17.0-52.0)	28.6 (16.0-52.0)	23.2 (13.0-43.0)	22.8 (13.0-42.0)
Ferritin <15 µg/L (%)[4]	664 (17.2%)	690 (17.8%)	845 (25.0%)	819 (24.5%)
Completed questionnaire	5154 (95.1%)	5133 (94.7%)	4650 (93.8%)	4672 (94.3%)
Gave blood sample	5293 (97.7%)	5312 (98.0%)	4798 (96.8%)	4816 (97.2%)

Data presented are mean (standard deviation) or number of participants (%) unless otherwise stated.
[1] Additional missing data: None for age, weight, or donation history; SF-36 Physical/Mental wellbeing scores (0.4% baseline, 6.4% end of main trial), haemoglobin (1.8% baseline, 2.6% end of main trial), ferritin (6.4% baseline, 10.5% end of main trial).
[2] A participant who has not previously provided a full blood donation.
[3] Total number (%) of deferrals or faints out of all attendances within 2-year period.
[4] Amongst those donating blood at baseline or end of main trial.
[5] Values are geometric means and interquartile ranges

eTable 7. Blood donations and other outcomes during the extension study by and reminder approaches.

	Men (N = 10843 randomised)			Women (N = 9914 randomised)		
Outcomes	**Routine**	**Active**	**P-value[6]**	**Routine**	**Active**	**P-value[6]**
No (% out of total in extension)[1]	5420 (50%)	5423 (50%)	-	4958 (50%)	4956 (50%)	-
Follow up time (yrs), median (IQR)	1.2 (0.8-1.3)	1.1 (0.8-1.3)	-	1.1 (0.6-1.2)	1.1 (0.6-1.3)	-
Blood donation rate (times/yr)	3.39 (3.34, 3.44)	3.50 (3.45, 3.54)	0.0003	2.28 (2.24, 2.31)	2.33 (2.30, 2.37)	0.009
Deferral for low Hb (%)[2]	4.64 (4.34, 4.95)	4.64 (4.34, 4.94)	0.878	5.48 (5.05, 5.91)	5.29 (4.87, 5.71)	0.435
Deferral for other reasons (%)[2]	3.49 (3.23, 3.76)	3.65 (3.39, 3.91)	0.378	4.77 (4.38, 5.16)	4.87 (4.48, 5.26)	0.748
Fainting at donation session (%)[2]	0.17 (0.11, 0.23)	0.16 (0.11, 0.21)	0.747	0.47 (0.34, 0.60)	0.50 (0.37, 0.63)	0.854
SF-36 Physical wellbeing score	56.5 (56.3, 56.6)	56.5 (56.4, 56.7)	0.548	56.5 (56.3, 56.7)	56.4 (56.2, 56.6)	0.164
SF-36 Mental wellbeing score	54.2 (54.1, 54.4)	54.1 (53.9, 54.3)	0.689	53.2 (52.9, 53.4)	53.1 (52.9, 53.3)	0.606
Haemoglobin (g/L)	142.6 (142.3, 142.9)	142.6 (142.2, 142.9)	0.793	130.9 (130.6, 131.2)	130.9 (130.6, 131.2)	0.547
Haemoglobin <135 g/L (men) or <125 g/L (women) (%)[3]	16.88 (15.57, 18.18)	17.52 (16.19, 18.85)	0.697	19.34 (17.87, 20.81)	18.79 (17.31, 20.26)	0.617
Ferritin (µg/L)[4]	30.7 (29.8, 31.5)	30.0 (29.2, 30.8)	0.868	25.8 (25.1, 26.5)	24.9 (24.2, 25.7)	0.072
Ferritin <15 µg/L (%)[3]	15.62 (14.27, 16.97)	16.67 (15.28, 18.06)	0.457	19.95 (18.37, 21.53)	22.24 (20.57, 23.91)	0.034
Serious adverse events (%)[5]	2.69 (2.24, 3.15)	2.63 (2.18, 3.08)	0.880	3.39 (2.86, 3.93)	2.88 (2.39, 3.38)	0.174

Data presented are mean or percentage (95% CI) unless otherwise stated.

[1] Additional missing data during the extension study were: <0.2% for blood donation, deferrals, or fainting; 21.0% for SF-36 Physical/Mental wellbeing scores, 25.0% for haemoglobin, 34.1% for ferritin. Higher SF-36 scores indicate better physical/mental wellbeing (0-100 scale range).

[2] Deferral or fainting rate per donation session attended during the extension study.

[3] Amongst those donating blood at end of extension study.

[4] Values are geometric means.

[5] Percentage of participants reporting any serious adverse events during the extension study, in any of the 6-monthly questionnaires: including doctor-confirmed heart failure, heart attack, angina, stroke, or transient ischaemic attack; hospital visit for falls or transport accidents

[6] P-values are for group differences, from analyses adjusted for baseline characteristics (centre, age, weight, new donor status) and value of the outcome at baseline (where available) and main trial randomised group (as linear trend).

eTable 8. Reported symptoms during the extension study by inter-donation intervals.

	**Men (N = 10843 in extension)**				**Women (N = 9914 in extension)**			
Outcomes	**8 weeks**	**10 weeks**	**12 weeks**	**P-value[3]**	**12 weeks**	**14 weeks**	**16 weeks**	**P-value[3]**
No (% out of total in extension)[1]	3554 (33%)	3695 (34%)	3594 (33%)	-	3369 (34%)	3309 (33%)	3236 (33%)	-
**Reported 6-monthly[2]**								
Fainting or feeling faint (%)	5.74 (5.09, 6.38)	4.99 (4.40, 5.57)	4.70 (4.11, 5.28)	0.018	8.29 (7.49, 9.08)	7.59 (6.84, 8.35)	7.79 (6.99, 8.59)	0.426
More tired than usual (%)	16.64 (15.59, 17.68)	15.08 (14.09, 16.06)	15.19 (14.18, 16.20)	0.065	20.29 (19.10, 21.47)	19.26 (18.09, 20.43)	20.08 (18.87, 21.29)	0.868
More breathless than usual (%)	5.00 (4.38, 5.61)	4.67 (4.08, 5.26)	4.02 (3.48, 4.56)	0.016	5.53 (4.88, 6.18)	4.69 (4.08, 5.30)	4.92 (4.29, 5.56)	0.181
Palpitations (%)	5.42 (4.75, 6.09)	5.58 (4.91, 6.26)	5.26 (4.60, 5.92)	0.873	11.69 (10.68, 12.71)	9.93 (8.99, 10.87)	11.23 (10.23, 12.24)	0.611
Dizziness (%)	8.84 (8.02, 9.66)	8.22 (7.45, 8.99)	7.55 (6.82, 8.29)	0.016	13.87 (12.84, 14.90)	13.03 (12.02, 14.03)	13.05 (12.03, 14.08)	0.300
Chest pain (%)	3.19 (2.71, 3.66)	3.19 (2.71, 3.68)	2.91 (2.45, 3.37)	0.425	2.47 (2.03, 2.90)	2.50 (2.06, 2.93)	2.67 (2.19, 3.14)	0.501
Restless legs (%)	14.55 (13.46, 15.64)	14.25 (13.18, 15.33)	13.92 (12.84, 14.99)	0.362	20.67 (19.33, 22.01)	20.45 (19.10, 21.81)	20.09 (18.74, 21.45)	0.500
Doctor diagnosed low iron levels (%)	6.60 (5.94, 7.27)	4.35 (3.82, 4.88)	3.39 (2.91, 3.88)	<0.0001	6.08 (5.41, 6.75)	5.66 (5.02, 6.30)	4.59 (3.99, 5.19)	0.002
Doctor prescribed iron supplements (%)	4.01 (3.48, 4.54)	2.50 (2.09, 2.92)	2.29 (1.88, 2.70)	<0.0001	4.57 (3.97, 5.17)	4.46 (3.86, 5.05)	3.53 (3.00, 4.06)	0.014
Pica (%)	0.79 (0.52, 1.07)	0.63 (0.42, 0.83)	0.68 (0.44, 0.91)	0.810	1.82 (1.41, 2.23)	1.64 (1.24, 2.04)	1.52 (1.13, 1.91)	0.357
**Reported at end of extension study**								
Any iron supplements (%)	19.83 (18.35, 21.31)	15.59 (14.28, 16.90)	13.40 (12.15, 14.65)	<0.0001	23.88 (22.25, 25.51)	21.22 (19.65, 22.79)	21.70 (20.10, 23.29)	0.063

Data presented are percentage (95% CI) unless otherwise stated.

[1] Additional missing data during the extension study were 11% for each symptom, and 21% for any iron supplements.

[2] Percentage of individuals reporting these symptoms at 6-monthly intervals, combining data from all the 6-monthly questionnaires during the extension study.

[3] P-values are for linear trend across groups, from analyses adjusted for baseline characteristics (centre, age, weight, new donor status).

eTable 9. Haematological markers at end of extension study by inter-donation intervals.

	Men (N = 10843 in extension)				Women (N = 9914 in extension)			
Outcomes	**8 weeks**	**10 weeks**	**12 weeks**	**P-value[2]**	**12 weeks**	**14 weeks**	**16 weeks**	**P-value[2]**
No (% out of total in extension)[1]	3554 (33%)	3695 (34%)	3594 (33%)	-	3369 (34%)	3309 (33%)	3236 (33%)	-
Red blood cell count, RBC ($x10^{12}/L$)	5.03 (5.02, 5.05)	5.05 (5.04, 5.07)	5.05 (5.03, 5.06)	0.0002	4.62 (4.61, 4.64)	4.62 (4.61, 4.63)	4.64 (4.63, 4.66)	0.0004
Haematocrit, HCT (%)	45.6 (45.5, 45.8)	46.1 (46.0, 46.3)	46.4 (46.3, 46.5)	<0.0001	42.7 (42.5, 42.8)	42.9 (42.7, 43.0)	43.0 (42.8, 43.1)	<0.0001
Mean corpuscular volume , MCV (fL)	90.9 (90.7, 91.2)	91.5 (91.3, 91.7)	92.2 (91.9, 92.4)	<0.0001	92.5 (92.2, 92.7)	92.9 (92.7, 93.2)	92.8 (92.6, 93.0)	0.046
Mean corpuscular haemoglobin, MCH (pg)	28.1 (28.0, 28.2)	28.3 (28.2, 28.4)	28.6 (28.6, 28.7)	<0.0001	28.2 (28.1, 28.4)	28.4 (28.3, 28.5)	28.5 (28.4, 28.6)	<0.0001
Mean corpuscular Hb concentration, MCHC (g/dL)	30.9 (30.8, 30.9)	30.9 (30.9, 31.0)	31.1 (31.0, 31.1)	<0.0001	30.5 (30.4, 30.6)	30.6 (30.5, 30.6)	30.7 (30.7, 30.7)	<0.0001
Reticulocyte Hb equivalent, RET-He (pg)	32.4 (32.3, 32.5)	32.7 (32.6, 32.8)	33.2 (33.1, 33.3)	<0.0001	32.3 (32.2, 32.4)	32.5 (32.4, 32.7)	32.8 (32.7, 32.9)	<0.0001
Platelet count, PLT ($x10^9/L$)	235 (233, 237)	235 (233, 237)	234 (232, 236)	0.107	269 (267, 271)	266 (263, 269)	267 (264, 269)	<0.0001
White blood cell count, WBC ($x10^9/L$)	6.07 (6.01, 6.13)	6.05 (6.00, 6.11)	6.05 (5.99, 6.10)	0.176	6.52 (6.45, 6.58)	6.48 (6.41, 6.54)	6.54 (6.47, 6.60)	0.695
Neutrophil count, NEUT# ($x10^9/L$)	3.50 (3.46, 3.55)	3.49 (3.44, 3.53)	3.48 (3.44, 3.53)	0.394	3.78 (3.73, 3.83)	3.76 (3.71, 3.81)	3.77 (3.72, 3.82)	0.728

Data presented are mean (95% CI) unless otherwise stated.
[1] Additional missing data during the extension study were 25% for each of the haematological markers.
[2] P-values are for linear trend across groups, from analyses adjusted for baseline characteristics (centre, age, weight, new donor status) and value of the outcome at baseline (where available).

eFigure 1. Extended CONSORT flow chart: recruitment, participation, and completeness of main outcomes in the extension study.

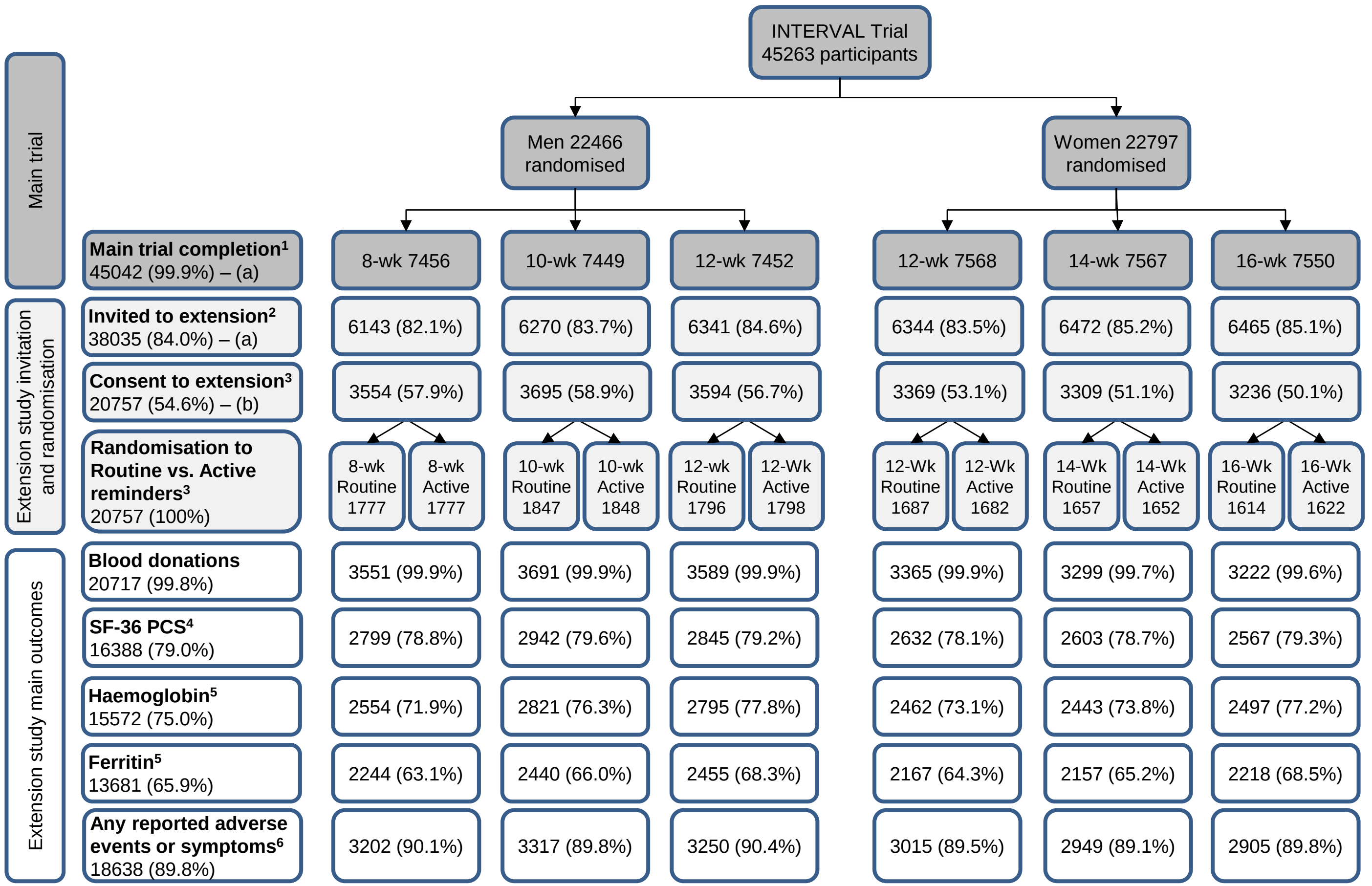

Centre	Main trial N	Extension n (Row %)
Leeds-City	2216	1037 (46.8%)
Bradford	1322	532 (40.2%)
Leeds-Sea	1039	494 (47.5%)
Sheffield	2614	1166 (44.6%)
Leicester	2053	867 (42.2%)
Nottingham	2580	1150 (44.6%)
Stoke	1340	576 (43.0%)
Birmingham	2184	1029 (47.1%)
Manchester-NH	2518	1054 (41.9%)
Cambridge	1798	992 (55.2%)
Brentwood[7]	756	1 (0.1%)
Lancaster	556	287 (51.6%)
Manchester-PG	1068	480 (44.9%)
Liverpool	2111	913 (43.2%)
Newcastle	1151	556 (48.3%)
Southmead-BR	1890	986 (52.2%)
Tooting	1488	689 (46.3%)
Southampton	865	501 (57.9%)
Oxford	1721	771 (44.8%)
Poole	1247	652 (52.3%)
Gloucester	438	268 (61.2%)
Plymouth	764	407 (53.3%)
Edgware	2098	1011 (48.2%)
Luton	1897	815 (43.0%)
West End	7328	3523 (48.1%)
Total	45042	20757 (46.1%)

Percentages are calculated with respect to numbers randomised in the main trial (a), or invited to the extension study (b), or otherwise randomised in the extension study.

[1] Excluding 221 participants who were randomised but later withdrew consent for any further use of their data.
[2] Due to staggered rollout of the main 2-year trial, only participants able to contribute at least 6 months of follow-up were considered eligible for invitation to the extension study.
[3] Participants not consenting to the extension study reverted to routine NHSBT management (men 12-wk, women 16-wk) and reminders.
[4] Number for whom a physical component wellbeing score (PCS) could be calculated at the end of the extension study.
[5] Number who provided a research blood sample at end of the extension study on which haemoglobin and ferritin were measured.
[6] Number who responded to at least one question in any of the 6-monthly questionnaires administered during their participation in the extension study.
[7] The Brentwood centre closed down after the main trial, but the single participant shown was donating elsewhere during the extension study.

eFigure 2. Whole blood donation rates during the extension study by sex and reminder approaches.

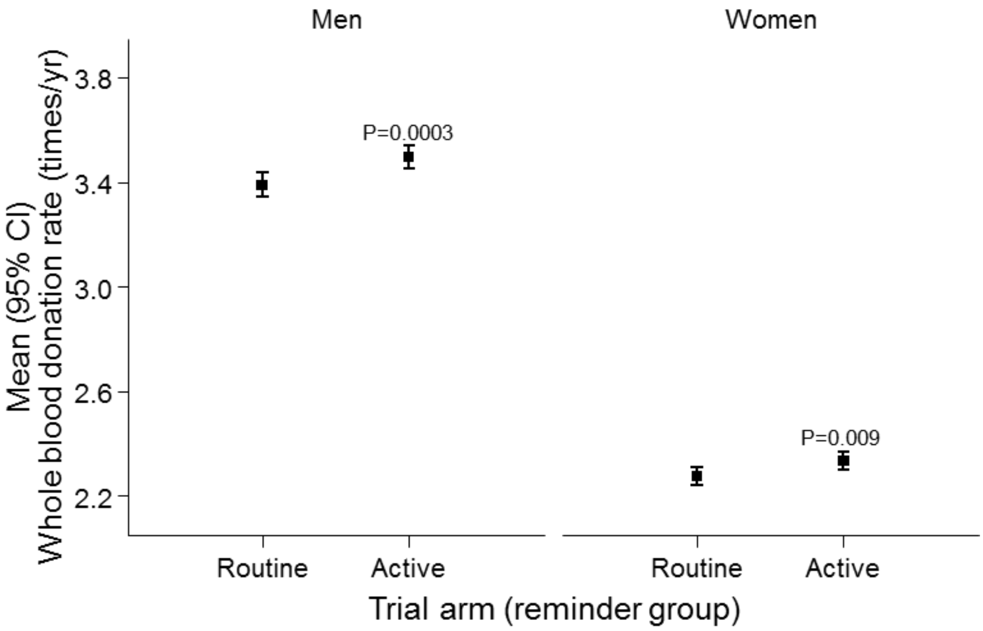

The p-values compare randomised groups adjusted for baseline characteristics (centre, age, weight, new donor status).

eFigure 3. Reported symptoms by sex and inter-donation intervals among participants in the extension study.

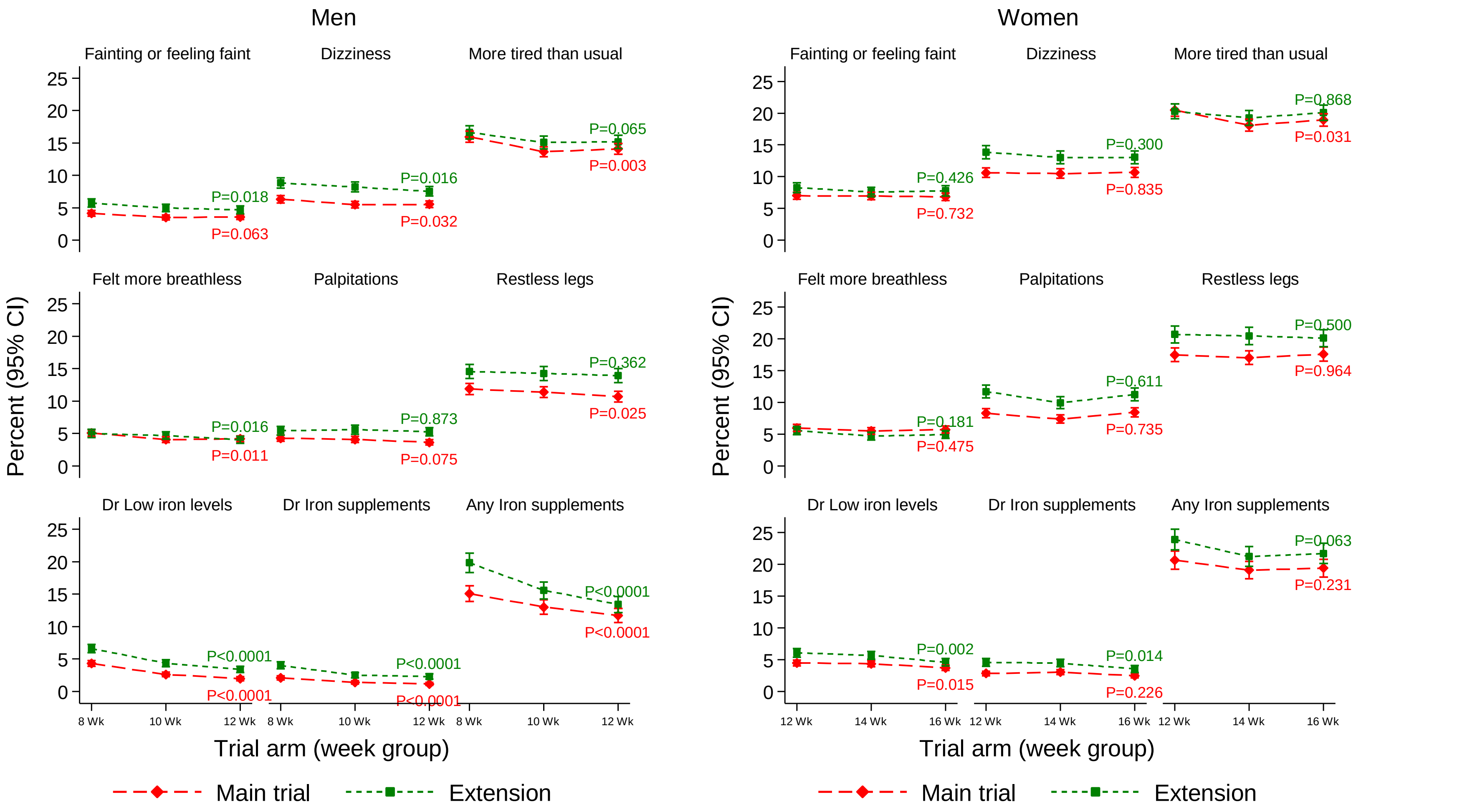

P-values assess trends across randomised groups, adjusted for baseline characteristics (centre, age, weight, new donor status).

eFigure 4. Low haemoglobin deferral rates by sex and inter-donation intervals among participants in the extension study.

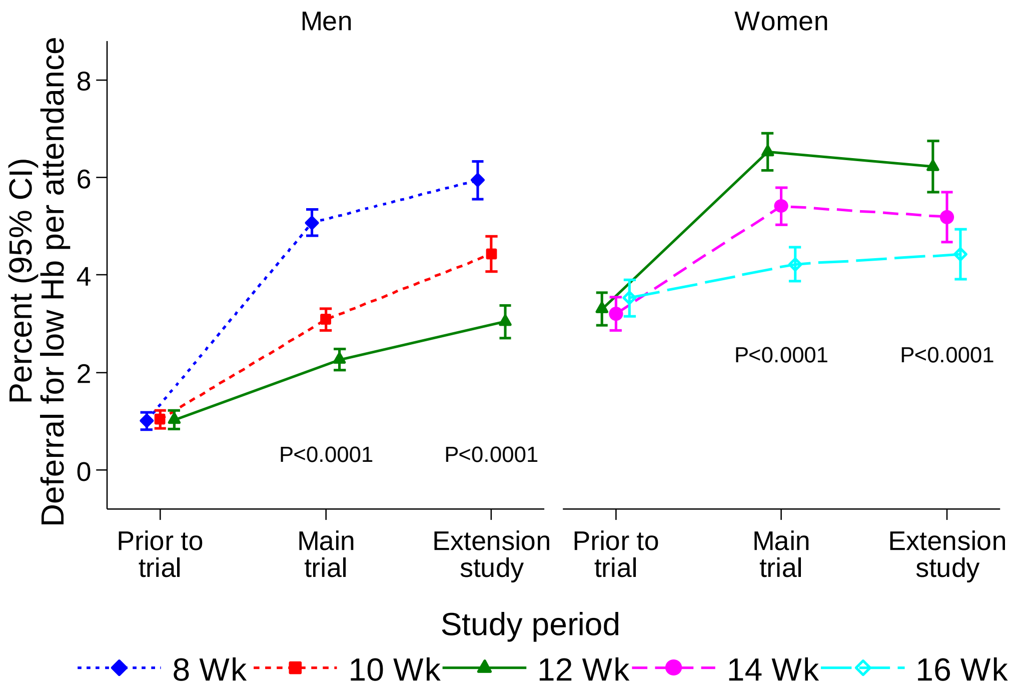

P-values assess trends across randomised groups, adjusted for baseline characteristics (centre, age, weight, new donor status).

eFigure 5. Haemoglobin concentrations below regulatory thresholds (a), and ferritin concentrations <15 µg/L (b), by sex and inter-donation intervals at the end of the extension study, end of the main trial period, and at baseline.

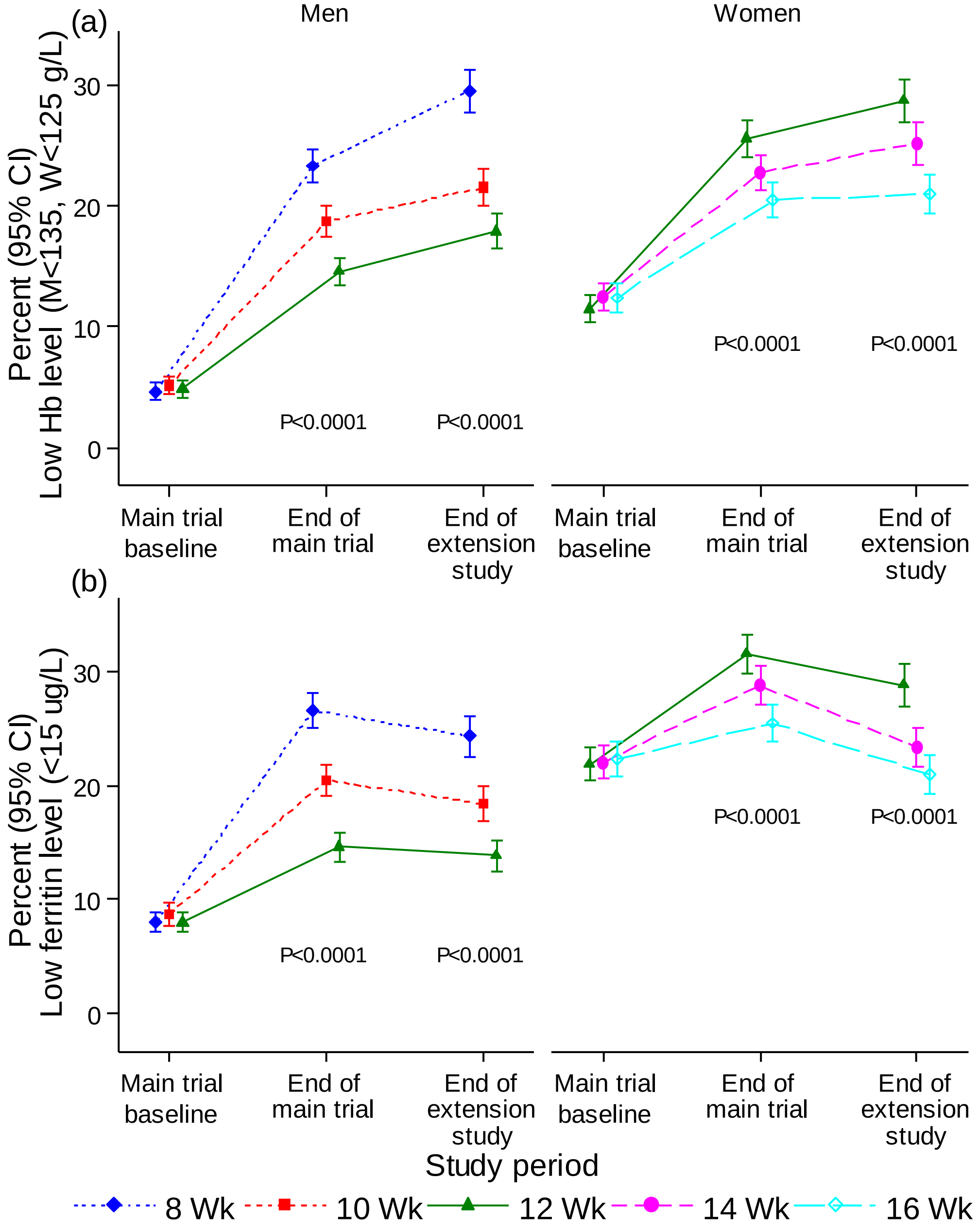

P-values assess trends across randomised groups, adjusted for baseline characteristics (centre, age, weight, new donor status, and low haemoglobin status (in a) or low ferritin status (in b)).

eFigure 6. Trends in standardized haematological characteristics by inter-donation intervals restricted to participants with ferritin measured at the end of the extension study.

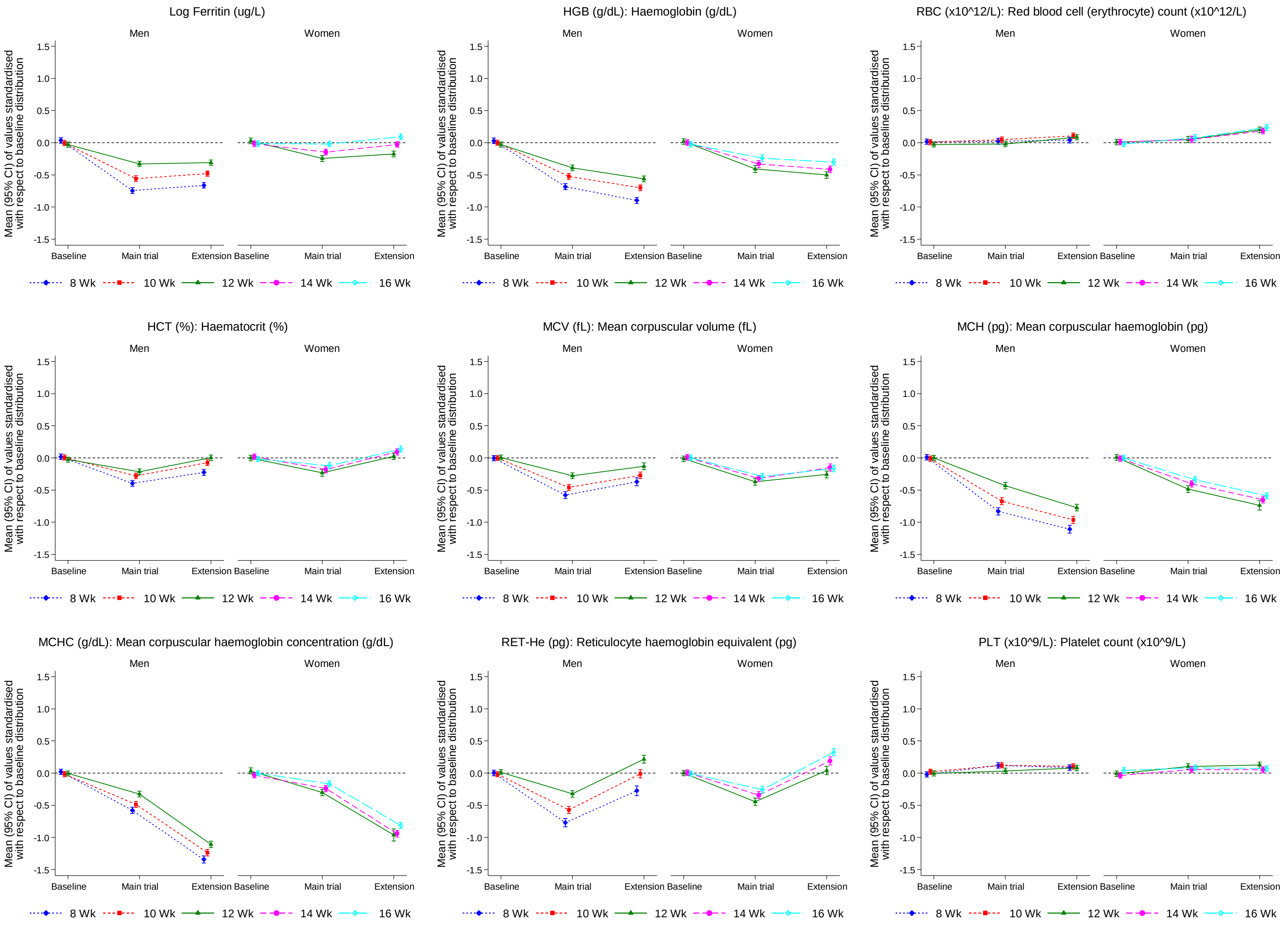

eFigure 7. Trends in reported use of iron supplements by inter-donation intervals among participants in the extension study.

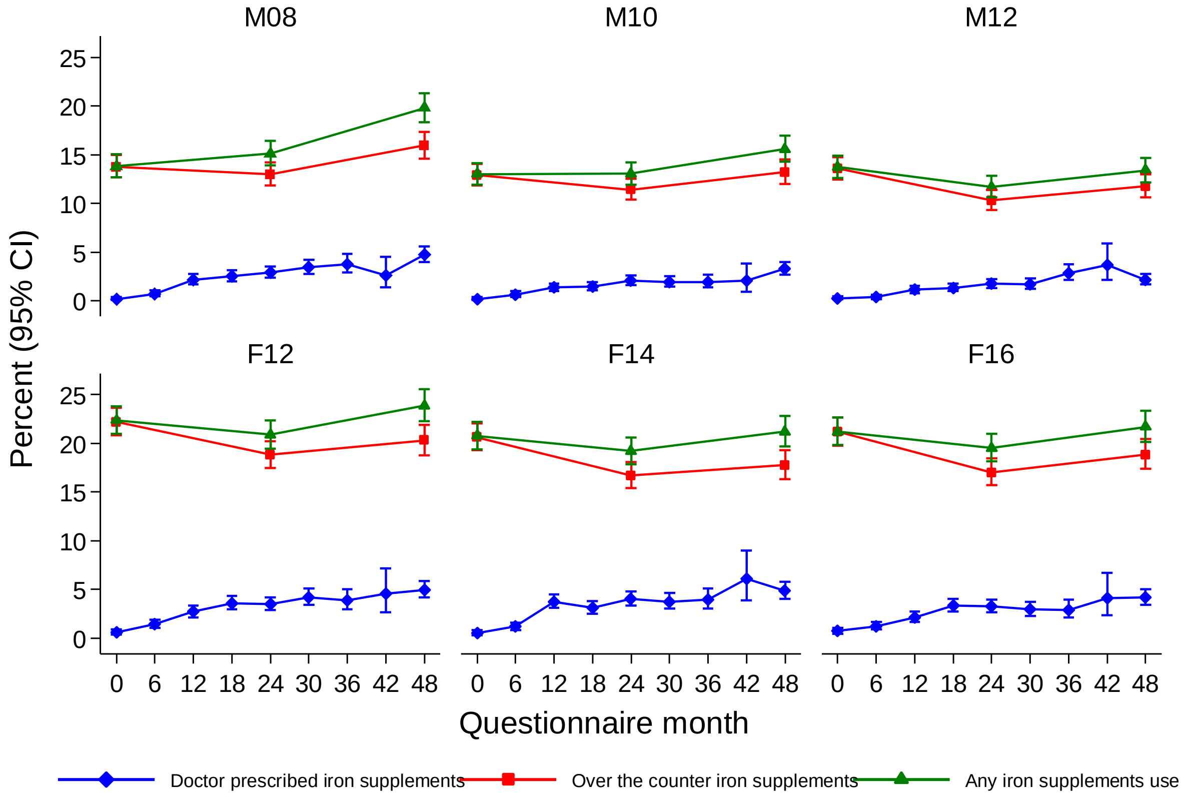

The randomised inter-donation week groups are abbreviated as M08, M10, M12 for men (top panels) and F12, F14, F16 for women (bottom panels).

eFigure 8. Haemoglobin concentrations by inter-donation intervals, stratified according to reported use of any iron supplements during the main trial or during the extension study, among participants in the extension study.

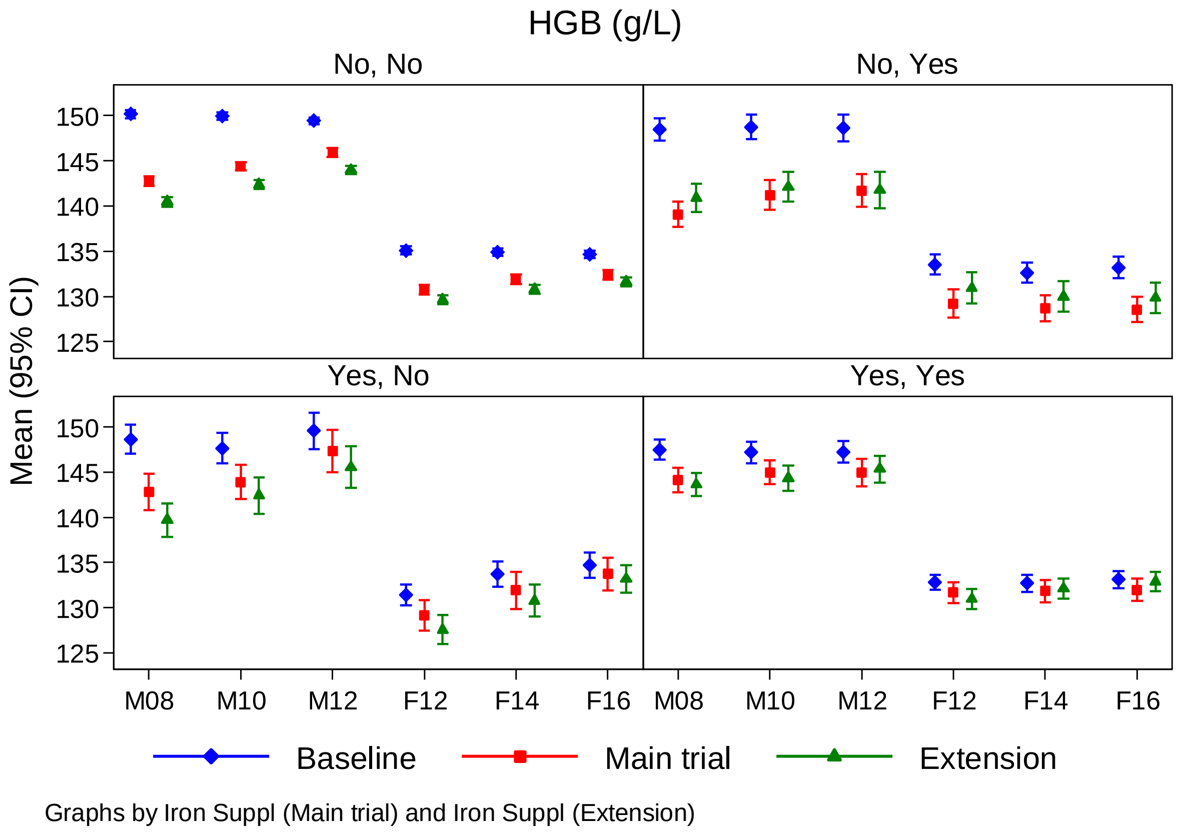

The randomised inter-donation week groups (x-axis) are abbreviated as M08, M10, M12 for men and F12, F14, F16 for women.

eFigure 9. Ferritin concentrations by inter-donation intervals, stratified according to reported use of any iron supplements during the main trial or during the extension study among participants in the extension study.

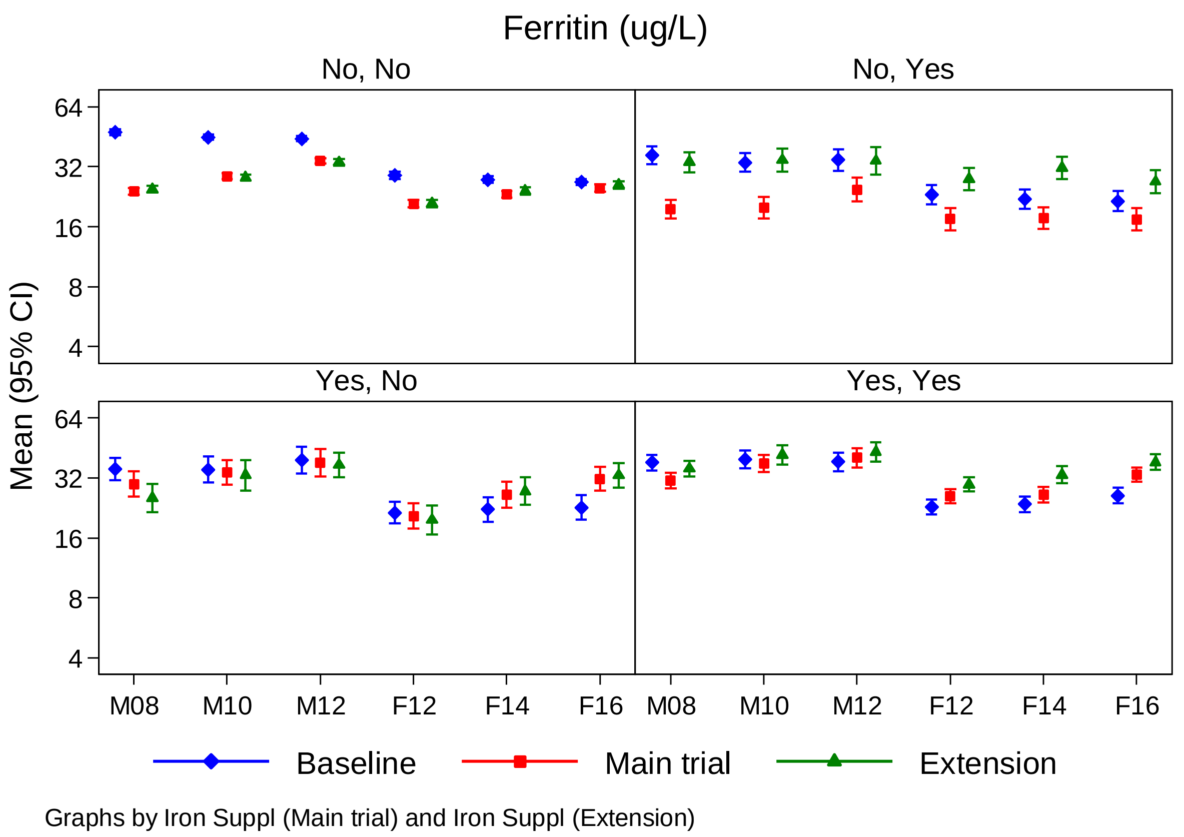

The randomised inter-donation week groups (x-axis) are abbreviated as M08, M10, M12 for men and F12, F14, F16 for women.

eFigure 10. Reticulocyte haemoglobin concentrations by inter-donation intervals, stratified according to reported use of any iron supplements during the main trial or during the extension study among participants in the extension study.

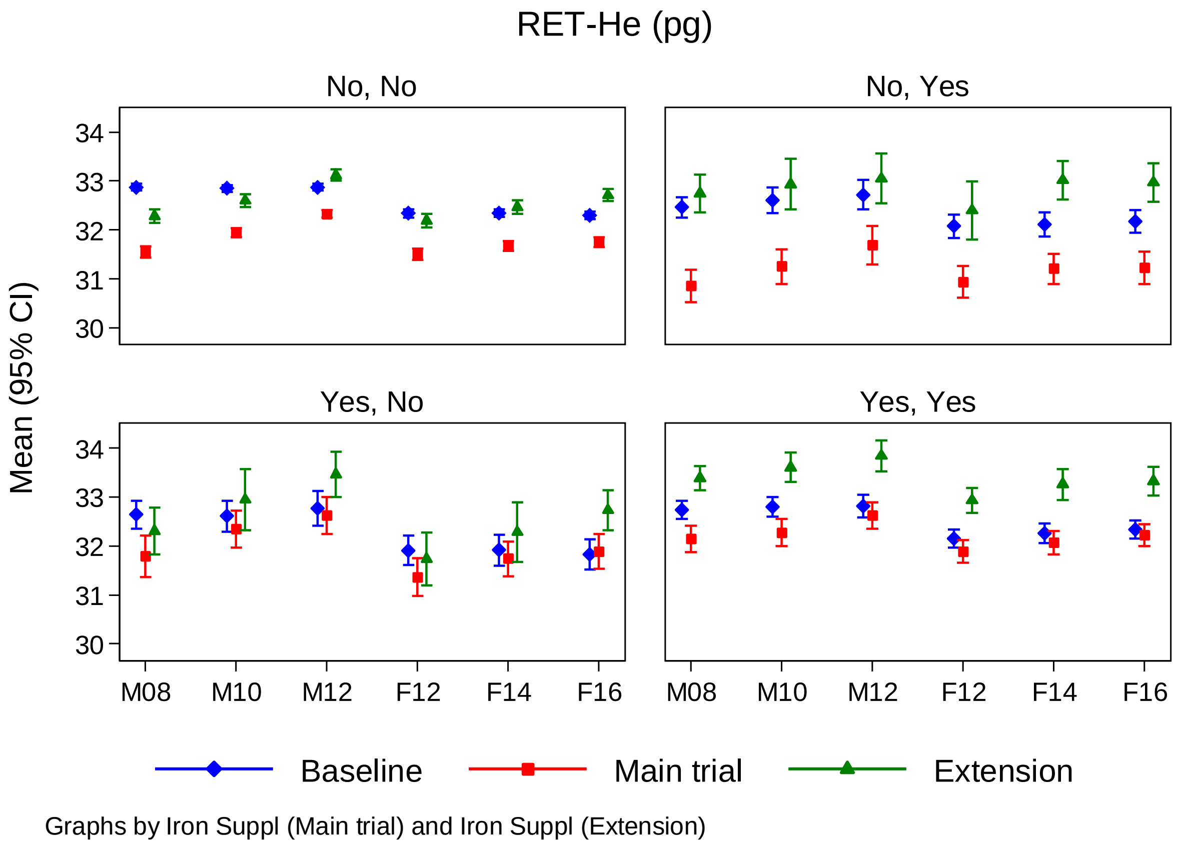

The randomised inter-donation week groups (x-axis) are abbreviated as M08, M10, M12 for men and F12, F14, F16 for women.

eAppendix

Extension study invitations, contributing data, and protocols for appointment reminders

Extension study invitations

Participants were eligible for further participation in the extension study immediately after completion of their 2-year participation in the main trial, provided they could contribute at least 6 months of follow-up before 15 June 2016. The end of main trial protocol included an email sent by the coordinating centre to alert donors of their 2-year anniversary date and procedures. This alert email was sent at the participant's 2-year anniversary date minus twice their allocated inter-donation interval (eg, at –16 weeks for 8-week group). A further reminder email was sent at 3 weeks prior to the 2-year anniversary date for those who had not made an appointment.

The design and ethical approval of the extension study was finalised after a few of the eligible participants had already completed their 2-year participation in the main trial, and after others had already received an email about their 2-year anniversary date. Therefore, the extension study invitations were either retrospective (ie, invited after the 2-year donation alert email had been sent) or prospective (ie, invited before the 2-year donation alert email had been sent). Active (ie, more intensive) reminders were used for all participants at the final planned attendance at the end of the extension study, to maximise the number of research blood samples taken at that time.

Data contributing to the extension study

Only data collected after randomisation in the extension study contributed to the data analysed. So for retrospectively recruited participants, there was a gap between the main trial and the extension study. Furthermore, for data derived from attendances for blood donation, the donation due at the end of the extension study was ignored, because all participants were more intensively reminded to attend this appointment, and follow-up was curtailed at the time the first reminder for this last appointment was sent. 30 days was allowed for completion of the 6-monthly online questionnaires, and up to the end of August 2016 for the final questionnaire.

Procedures to encourage donation attendance

The main INTERVAL trial used a more comprehensive approach (“active”) than that routinely used by NHSBT (“routine”) to remind participants to make and keep blood donation appointments (**eAppendix Figure**). First, the main trial’s reminder approach applied to all participants rather than, as in routine NHSBT practice, a subset of donors judged to be high priority by various criteria (eg, blood group, reliability, sex, recent contact). Second, participants in the main trial who had not made an initial donation appointment within 14 days

of randomisation received up to three reminders using a fixed protocol of telephone and/or email messages. Third, when a booking had not been made for a subsequent appointment three weeks prior to its due date, participants received an email reminder to book an appointment. Fourth, all participants received a text message reminder one day in advance of each appointment. Fifth, after failure to attend an appointment, participants were encouraged to make new appointments using the process described above for making an initial appointment after randomisation into the main trial. The NHSBT's routine reminder approach had some of the preceding features but differed in their timing and intensity of use.

eAppendix Figure

Donor with appointment			Donor without appointment			
NHSBT routine	INTERVAL - interim Appt	INTERVAL - last donation	NHSBT routine	INTERVAL - 1st Appt	INTERVAL interim Appt	INTERVAL last Appt
**-4w:** Reminder telephone call; either landline (57%) or SMS if mobile number available. SLA to contact 65% (this can be answerphone)		**-3w:** automated email reminder		**On randomisation:** automated email asking donors to contact ISAT to make next appointment	**At session:** Participants strongly encouraged to make appointment at donation attendance	**At session:** Participants strongly encouraged to make appointment at donation attendance
**-7-10d:** Appt letter & DHC	**-7-10d:** Appt letter & DHC	**-7-10d:** Appt letter & DHC		**+14 d No Appt - Step 1:** Telephone call & voicemail	**-3w No Appt:** Automated email to prompt donor to call ISAT	**-3w No Appt:** Automated email to prompt donor to call ISAT
**Week of Appt:** SMS (SMS reminders are stock-driven and 100% of donors receiving these email will receive all three reminders)	**-1d:** SMS	**-1d:** SMS	**-4w:** Sales call (generic voice mail), stock-driven, SLA 65%	**+21d No Appt - Step 2:** Telephone call & voicemail	**Day 0:** Appointment due date	**-7d No Appt - Step 1:** Telephone call & voicemail
**Day of Appt:** SMS	**Day 0:** Date of booked appointment	**Day 0:** Date of booked appointment	**-7d** Appt letter and DHC	**+28d No Appt - Step 3:** Email		**-6d-1d No Appt - Step 2:** Telephone call & voicemail
**Day of Appt:** Email reminder (added 2014)	**DNA - Step 1 :** Telephone call & voicemail	**DNA - Step 1 :** Telephone call & voicemail	**Day of session:** Telephone call & email, followed by SMS (if grid is 90% or less full)	**Day 0:** Appointment due date		**-6d-1d No Appt - Step 3:** Email
	**+7d DNA - Step 2 :** Telephone call & voicemail	**+7d DNA - Step 2 :** Telephone call & voicemail		**No Appt - Step 1 :** Telephone call & voicemail		**Day 0:** Appointment due date
	**+14d DNA - Step 3 :** Email	**+14d DNA - Step 3 :** Email		**+7d No Appt - Step 2 :** Telephone call & voicemail		**+2w No Appt - Step 1:** Telephone call & voicemail
		**+28d DNA - Step 1**: Telephone call & voicemail		**+14d No Appt - Step 3 :** Email		**+3w No Appt - Step 2:** Telephone call & voicemail
		**+35d DNA - Step 2**: Telephone call & voicemail				**+4w No Appt - Step 3:** Email
		**+42d DNA - Step 3**: Email				

Abbreviations: Appt Appointment; d day(s); DHC Donor Health Check (questionnaire); DNA did not attend; ISAT INTERVAL Study Administration Team; NHSBT National Health Service Blood and Transplant; SLA service level agreement; SMS short message service (ie, text message); w week(s)
